# A Sensitive Multichannel Fluorescent Polymer Sensor Array for the Detection of Protein Fluctuations in Serum

**DOI:** 10.3390/s26082308

**Published:** 2026-04-09

**Authors:** Junwhee Yang, Colby Alves, Kanwal Nazir, Mingdi Jiang, Nicolas Araujo, Vincent M. Rotello

**Affiliations:** Department of Chemistry, University of Massachusetts Amherst, 710 North Pleasant Street, Amherst, MA 01003, USA; junwheeyang@umass.edu (J.Y.); colbyalves1234@gmail.com (C.A.); knazir@umass.edu (K.N.); mingdijiang@umass.edu (M.J.); naraujo@umass.edu (N.A.)

**Keywords:** array-based sensing, chemical nose sensing, sensor array, serum diagnostics, protein identification, polymer

## Abstract

Serum contains diverse proteins whose concentrations vary with pathological conditions such as cancer, liver disease, neurological disorder, and infections. Conventional methods like serum protein electrophoresis (SPEP) and enzyme-linked immunosorbent assay (ELISA) are gold standards for protein identification; however, they are time-consuming and can miss abnormal serum protein levels. Inspired by chemical nose sensing based on selective sensor–analyte interactions, we synthesized five pyrene-conjugated fluorescent polymers (PFPs) with distinct side-chain head groups to construct a multichannel fluorescence sensor array. These polymers were screened for sensitivity to changes in serum protein levels using linear discriminant analysis (LDA), a machine learning method. This process led to the successful discovery of two PFPs that effectively detect protein level fluctuations. These PFPs provided a sensitive sensor array capable of generating a high-content response pattern (fingerprint) with six fluorescence channels. This sensor array successfully discriminated protein level fluctuations in serum with 98% jackknife classification accuracy and 95% unknown identification accuracy. This polymer sensor array holds strong potential as a diagnostic tool for serum-based samples and can be extended to other applications related to protein identification.

## 1. Introduction

Abnormal fluctuations in serum protein levels can reflect pathological conditions such as cancer [1,2,3], liver disease [4,5], neurological disorders [6,7], and viral infections [3,8]. Accordingly, monitoring changes in serum protein levels is crucial for early disease diagnosis and for tracking of disease progression [2,3,9]. It is essential to develop sensors capable of detecting subtle changes in serum protein concentrations to achieve these clinical objectives [10,11]. Serum protein gel electrophoresis (SPEP) and enzyme-linked immunosorbent assays (ELISA) are conventional methods for identifying serum protein levels. SPEP provides a straightforward approach for separating proteins based on their molecular weights, shapes, and charges. However, resolution and challenges in reproducibility limit the ability to detect subtle changes in serum protein levels [12,13,14]. ELISA offers a more sensitive protein quantification method by employing biomarker-specific antibodies. Nonetheless, serum is a complex mixture containing numerous proteins, with individual or multiple biomarkers often not providing diagnostic accuracy [15,16]. To address these challenges, several biosensors using aptamers and antibody-conjugated nanomaterials have been proposed that enable sensitive protein detection in serum [15,17,18,19]. While these approaches enhance sensitivity, they still rely on specific interactions with target proteins, which can limit diagnostic accuracy in serum due to undesired cross-reactivity with non-target molecules [20,21].

“Chemical nose” sensing offers an alternative to conventional biomarker-specific strategies for profiling complex mixtures such as serum [22,23]. This approach uses sensor elements that interact *selectively* with multiple components in the analyte, generating unique response patterns (or “fingerprints”) that can discriminate between complex analyte mixtures with subtle differences [20,21,23,24]. Polymer sensor arrays have emerged as powerful tools for measuring protein levels in vitro and ex vivo [25,26,27]. Previously reported polymer sensor arrays have enabled high-throughput detection of protein level changes but relied on a single recognition element. Although these approaches have demonstrated potential applications of polymer sensor arrays as cancer and liver fibrosis diagnosis, enhancing sensitivity of the sensor platform still remains critical for clinical translation.

**Figure 1:**
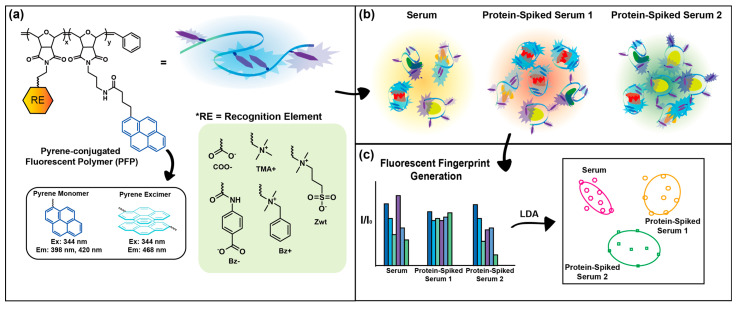
A schematic illustration of the process for detecting protein level changes in serum. (**a**) Pyrene-conjugated fluorescent polymers (PFPs) featuring different side-chain head groups. (**b**) Spontaneous and selective interactions between polymers and proteins in serum. (**c**) Generation of distinctive fluorescent recognition patterns for each serum analyte, and detection of protein concentration changes using linear discriminant analysis (LDA). Arrows between panels indicate the sequential workflow from polymer sensor design to serum analyte discrimination.

Here, we hypothesize that sensor sensitivity can be enhanced by expanding polymer–protein interaction modes through the incorporation of chemically distinct recognition elements and systematic screening of their signal responses (Figure 1). To test the hypothesis, we synthesized five pyrene-conjugated fluorescent polymers (PFPs) bearing different side chains to construct multichannel fluorescent polymer sensor arrays for sensitive and high-throughput detection of protein level fluctuations in serum. Specifically, each PFP was functionalized with one recognition element: trimethyl quaternary ammonium (TMA+), carboxylate (COO−), alkyl benzyl dimethyl quaternary ammonium (Bz+), benzoate (Bz−), or zwitterionic (Zwt) moieties. Pyrene was chosen as the transducing element and conjugated to the polymers due to its ability to generate multiple fluorescent signals, thereby providing multi-channel outputs [27,28]. We conducted sensing experiments with these five polymers to evaluate their contributions to the discrimination between control serum and protein-spiked sera. Human serum albumin (HSA) [29], fibrinogen (Fib) [30], immunoglobulin G (IgG) [31], and transferrin (Trf) [32] were selected for their clinical relevance across multiple disease states. We identified PFP (Bz−) and PFP (Bz+) as key contributors to discriminating protein-level perturbations in serum through systematic screening. We achieved 98% classification accuracy and 95% unknown identification accuracy by combining fluorescent signals from PFP (Bz−) and PFP (Bz+), demonstrating the platform’s sensitivity and reproducibility. This promising result indicates that the sensitivity of the sensor can be improved by using multiple sensor molecules with different recognition elements, providing a pathway for constructing array-based sensors for serum diagnosis and protein identification in complex mixtures for broader applications.

## 2. Materials and Methods

### 2.1. Materials

All chemicals and proteins used in this article were purchased from Sigma-Aldrich (St. Louis, MO, USA), Acros Organics (Geel, Belgium), and Tokyo Chemical Industry Co., Ltd. (TCI; Tokyo, Japan). Dulbecco’s phosphate-buffered saline (DPBS) without calcium and magnesium was used for preparing protein and polymer stock solutions for the model serum protein binding study, protein-spiked serum discrimination experiments, and analyses. Fetal bovine serum (FBS) from Gibco was used as the model serum, and all experiments and analyses related to protein-spiked serum discrimination were conducted using it.

### 2.2. Synthesis and Characterization

Monomers used for polymerization were synthesized as described in previously reported studies [24,25,33,34]. The PFP (Zwt) and polymer backbones used for preparing the other four PFPs were first synthesized via ring-opening metathesis polymerization (ROMP). Typically, 100 mg of the oxanorbornene monomer (95 mol%) bearing reactive sites for post-functionalization of side-chain head groups was added to a 10 mL pear-shaped Schlenk flask and dissolved in 3 mL of dichloromethane (DCM). In the case of the Zwt-functionalized monomer, 3 mL of trifluoroethanol/DCM (2:1) was used. Subsequently, the pyrene-conjugated oxanorbornene monomer (5 mol%) was dissolved in 1 mL of DCM and added to the same Schlenk flask. Afterwards, 4 mg of the third-generation Grubbs catalyst was weighed, added to another 10 mL Schlenk flask, and dissolved in 1 mL of DCM. Both solutions were degassed using the freeze–pump–thaw method until no bubbles were observed. Subsequently, the catalyst solution was transferred to the flask containing the monomer solution. The reaction mixture was vigorously stirred under nitrogen for 20 min, after which 200 µL of ethyl vinyl ether was added, and the mixture was stirred for an additional 15 min. The crude product was directly eluted through a short pad of aluminum oxide, concentrated using a rotary evaporator, and precipitated twice in an ethyl ether/hexane (1:1–1:4) mixture to obtain the polymer backbones in yields greater than 90%. The reaction schemes for the polymer backbones and PFP (Zwt), and their characterizations, are included in the Supporting Information (SI, Figures S1–S5). Detailed procedures for the synthesis of PFPs and their characterization are also provided in the supporting information (SI, Figures S6–S10).

### 2.3. Dynamic Light Scattering (DLS) and Zeta Potential Measurement

A Malvern Zetasizer Nano ZS (Malvern Instruments; Malvern, UK) was used to measure the hydrodynamic sizes and surface charges of PFPs. For DLS, a 10 mg/mL stock solution of PFP was diluted to 1 mg/mL in DPBS without calcium and magnesium and transferred to a disposable cuvette for size measurement. The hydrodynamic sizes of all PFPs were reported as number-weighted distributions to assess the dominant polymer population. For polymer assembly behavior studies, polymer solutions were mixed with HSA and incubated for 30 min prior to measurement. The mixed solutions contained 50 µg/mL PFPs and 0.2, 0.5, or 1 mg/mL HSA. Intensity-weighted size distributions were reported to monitor changes in polymer assembly. For zeta potential measurements, the PFP stock solution was diluted to 50 µg/mL in 10 mM NaCl and added to a zeta potential cell for charge measurement.

### 2.4. Observation of the Absorbance and Fluorescence Spectra of PFPs

Each polymer was diluted in DPBS to a concentration of 100 µg/mL, and 1 mL of each solution was transferred into 1.5 mL disposable UV cuvettes. After recording a reference spectrum using a cuvette containing blank DPBS, the polymer solutions were scanned from 300 to 550 nm at 2 nm intervals to obtain the absorbance spectrum, and from 360 to 600 nm at 2 nm intervals to obtain fluorescence spectra. The same procedure was applied to acquire fluorescence spectra for all five polymers. A SpectraMax M2 plate reader (Molecular Devices; San Jose, CA, USA) was used for this study.

### 2.5. Model Protein Titration Study

All polymers were diluted in DPBS to a concentration of 55.6 µg/mL, and 90 µL of each polymer solution was loaded into individual wells of a 96-well microplate. Subsequently, 10 µL of HSA in DPBS was added to the wells containing polymer solutions. The final concentration of the polymer was 50 µg/mL, and the final concentration of HSA ranged from 0 to 5 mg/mL. After a 45 min incubation at room temperature in the dark, fluorescence spectra were recorded using a SpectraMax M2 plate reader (Molecular Devices; San Jose, CA, USA) at an excitation wavelength of 344 nm, with measurements taken at 2 nm intervals.

### 2.6. Sensing Experiment

For all protein-spiked serum discrimination experiments, 90 µL of a 55.6 µg/mL polymer solution was added to each well of a 96-well plate, followed by the addition of 10 µL of serum analyte. This yielded a final polymer concentration of 50 µg/mL. As an experimental control, DPBS was added to the polymer solution instead of serum analytes to establish the reference fluorescence (I_0_). After incubation for 45 min at room temperature in the dark, all mixed solutions were excited at 344 nm, and fluorescence emissions at 398 nm (Py Monomer 1), 420 nm (Py Monomer 2), and 474 nm (Py Excimer) were measured using a SpectraMax M2 plate reader (Molecular Devices; San Jose, CA, USA). Fluorescence responses (I) from each well containing polymer–serum mixtures were normalized by dividing by the fluorescence intensity of the DPBS reference (I_0_), yielding I/I_0_ values for fluorescence pattern generation.

### 2.7. Linear Discriminant Analysis (LDA)

We applied LDA on normalized fluorescence responses (I/I_0_) to statistically classify serum analytes using SYSTAT version 11.0 (SystatSoftware) [35,36]. The tolerance was set to 0.001, and the input data, the raw fluorescence responses (I/I_0_), were transformed into canonical scores, with the types of proteins spiked into serum used as the grouping variable. After the data transformation, LDA reduced the dimensionality of the data and projected the discrimination result in a two-dimensional scatterplot with a 95% confidence ellipse. All analyses were performed using eight replicate measurements of fluorescence responses (I/I_0_) obtained from the sensing experiment for each analyte group.

### 2.8. Jackknife Classification

Jackknife classification is a cross-validation method that helps validate the accuracy of the classification [37]. In this analytical method, one sample observation is omitted from the dataset, generating n subsets, each containing n–1 samples. The samples in each subset are used as training sets to create discriminant functions for classifying the left-out samples. The overall classification accuracies for the left-out sample classification are then computed to obtain the average classification accuracy. The Jackknife classification was presented along with the classification results the scatterplot to demonstrate the discrimination quality and reproducibility of all sensor arrays.

### 2.9. Unknown Identification

The identities of blind serum analytes were predicted by calculating Mahalanobis distances (defined as the distance between a data point and a distribution center) between unknown samples and the centroids of training groups in the two-dimensional classification matrix generated by LDA [35]. The normalized fluorescence responses (I/I_0_) of 40 blind serum analytes were transformed into canonical scores using the discriminant functions established from the training set used for protein-spiked serum discrimination. Each blinded serum sample was then predicted to the closest training group in the two-dimensional classification matrix, as determined by the shortest Mahalanobis distance.

## 3. Results and Discussion

### 3.1. Polymer Synthesis

The backbones of all PFPs were synthesized via ring-opening metathesis polymerization (ROMP), employing oxanorbornene imide-based monomers and a third-generation Grubbs’ catalyst (Figure S1). ROMP was selected as the polymerization method due to its ability to yield polymers with highly controlled molecular weights and low polydispersity indexes (PDIs) [38,39]. The poly(oxanorbornene) imide (PONI) polymers are reported to possess semi-rigid backbone structure [40,41,42]. We assumed that this semi-rigid backbone may favor local conformational changes upon analyte binding, rather than large-scale reorientation of polymer chain. We anticipated that these local perturbations would lead to more stable and reproducible signal responses in polymer-based sensor platforms. As described in Figure 1a and Figures S1–S9, the side chains of the polymers were functionalized either before or after polymerization with distinct head groups (COO−, Bz-, TMA+, Bz+, and Zwt). These head groups were selected to facilitate spontaneous interactions—electrostatic, hydrophobic, π–π stacking, and π–charge interactions—between the polymers and serum proteins. We incorporated different functional groups as recognition elements to enable the discovery of highly sensitive polymer sensor components. As mentioned above, pyrene was selected as the transducing element due to its multiple fluorescence channels arising from pyrene monomer and excimer signals. Relative fluorescence intensities of pyrene monomers and excimer were strongly governed by the proximity of pyrene units conjugated at the polymer backbone. We hypothesized that PFP–protein interactions would perturb the local polymer conformation. This perturbation, in turn, alters the emission balance between pyrene monomer and excimer states, as well as the quenching–dequenching behavior of the overall signal [27,28]. Multi-dimensional information with a minimal number of sensor units enables the facile formulation of a high-throughput and high-content sensor, allowing for sensitive analyte profiling [43].

### 3.2. Polymer Characterization

**Figure 2:**
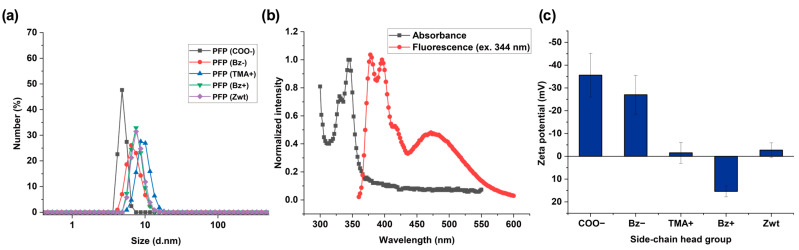
(**a**) Hydrodynamic sizes of PFPs obtained by DLS (*n* = 3): PFP (COO−), 4.9 ± 0.6 d.nm; PFP (Bz−), 8.1 ± 1.7 d.nm; PFP (TMA+), 10.2 ± 1.8 d.nm; PFP (Bz+), 7.7 ± 1.5 d.nm; and PFP (Zwt), 8.1 ± 1.7 d.nm. (**b**) Normalized absorbance and fluorescence spectra of PFP functionalized with Bz− (*n* = 3). (**c**) Zeta potentials of PFPs functionalized with varying side-chain head groups (Error bars: ± S.D., *n* = 3).

Serum is an aqueous solution that is highly concentrated with various protein species [12,44]. We hypothesized that undesirable aggregation of polymer sensors in aqueous solution at physiological pH would hinder efficient polymer–serum protein interactions, thereby reducing the sensitivity of signal changes from the PFPs. Thus, we targeted low-molecular-weight polymers (<30 kDa) to minimize aggregation that can occur in high-molecular-weight polymers [45,46]. Both the number-average molecular weights (M_n_) and the weight-average molecular weights (M_w_) of all polymer backbones used for synthesizing PFPs were 20–30 kDa as demonstrated by gel permeation chromatography (GPC) (Figure S10). A total of 1 mg/mL of each PFP was dissolved in pH 7.4 DPBS and analyzed by DLS to measure their hydrodynamic sizes to observe whether the PFPs form significant aggregates in aqueous solution. As shown in Figure 2a, the hydrodynamic sizes of all PFPs were approximately 5–10 nm, indicating that the PFPs were not susceptible to aggregation in aqueous solution at physiological pH. To verify successful incorporation of pyrene into the polymers, we recorded absorbance and fluorescence spectra of PFP functionalized with Bz− (Figure 2b). As expected from previously reported results, we observed both pyrene monomer and excimer fluorescence [28], spanning from 360 nm to 600 nm when excited at 344 nm. To examine the surface charges of all PFPs, zeta potential measurements were conducted. As shown in Figure 2c, the zeta potential values were −35.6 ± 9.6 mV for PFP (COO−), −27.0 ± 8.5 mV for PFP (Bz−), −1.5 ± 4.6 mV for PFP (TMA+), 11.0 ± 7.5 mV for PFP (Bz+), and −2.7 ± 3.2 mV for PFP (Zwt), respectively.

### 3.3. Fluorescence Responsiveness Screening of Polymers Toward a Model Serum Protein

**Figure 3:**
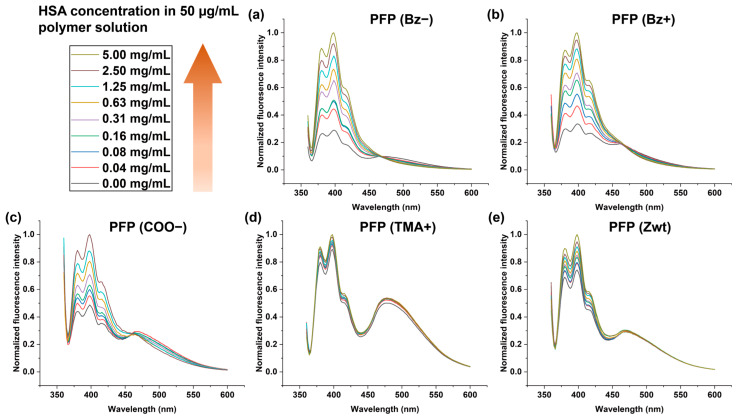
Changes in the fluorescence spectra of each polymer upon titration with increasing concentrations of HSA (*n* = 3). Fluorescence spectral changes in (**a**) PFP (Bz−), (**b**) PFP (Bz+), (**c**) PFP (COO−), (**d**) PFP (TMA+), and (**e**) PFP (Zwt).

We selected human serum albumin (HSA) as a model protein to screen the responsiveness of polymer sensors, as it is one of the major serum components and is clinically associated with liver cirrhosis [47], kidney dysfunction [29], and diabetes [48]. We predicted that fluorescence emission wavelengths displaying the most fluorescence responses (I/I_0_) upon addition of HSA to the polymer solutions would contribute to the formulation of fluorescence patterns with optimal sensitivity. Therefore, fluorescence emissions at 398 nm (pyrene monomer 1), 420 nm (pyrene monomer 2), and 474 nm (pyrene excimer) were chosen for generating fluorescence fingerprints as they exhibited the most dynamic signal changes. As shown in Figure 3a–e, increasing the concentration of HSA induced greater signal changes across most polymers.

Notably, PFPs with aromatic side-chain head groups or high surface charges (PFP (Bz−) and PFP (Bz+)) exhibited more pronounced spectral responses (Figure 3a,b). In contrast, PFP (TMA+) and PFP (Zwt), which possess near-neutral zeta potentials in DPBS (Figure 2c), exhibited negligible fluorescence changes upon HSA titration (Figure 3d,e). These observations imply that the surface charge and aromaticity of PFPs play a crucial role in how the local polymer environment is perturbed upon protein addition, thereby influencing the dynamic range of the fluorescence response (I/I_0_). To further understand the underlying mechanism of these dynamic fluorescence changes, DLS experiments were performed for PFP (Bz−) and PFP (Bz+). As shown in Figure S11, increasing the concentration of HSA in both polymer solutions led to changes in assembly size distributions (intensity-weighted) compared to polymer-only samples. These results indicate that protein addition alters polymer assembly behavior, which likely contributes to the observed changes in pyrene emission.

### 3.4. Identification of Key Contributors for Detecting Protein Level Changes in Serum

**Figure 4:**
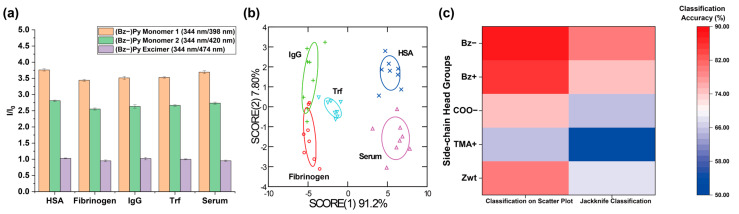
Classification of protein-spiked sera and the control serum using PFPs. (**a**) Fluorescence bar graph obtained from 50 μg/mL PFP (Bz−) after incubation with serum samples for 45 min (*n* = 8). (**b**) LDA plot of the fluorescence patterns generated from serum–PFP (Bz−) interactions. (**c**) Heatmap showing LDA classification accuracies for PFPs functionalized with different side-chain head groups.

We first screened the sensitivity of five different polymers in detecting protein-level changes in serum before constructing the polymer sensor array. Four clinically relevant serum proteins (HSA, Fib, IgG, and Trf) were selected and spiked into 10% FBS at 1 mg/mL. FBS was chosen as a model serum to create a controlled and reproducible protein-spiked serum system. Given that each PFP generates multidimensional signal outputs, reducing dimensionality is crucial for both efficient pattern recognition and efficient analyte discrimination [49]. Linear discriminant analysis (LDA) is a supervised dimensionality reduction and classification tool that maximizes the separation between different classes and enables accurate classification for blind analytes [35,36]. LDA was therefore performed to discover polymers with high classification accuracies for serum analyte identification. The three fluorescence responses (I/I_0_) determined from the HSA titration study were utilized as the input data set (8 replicates per serum sample) for LDA. As demonstrated in Figure 4 and Figure S12, PFP (Bz−) and PFP (Bz+) exhibited the highest classification accuracies in both the two-dimensional classification matrix (92% for PFP (Bz−) and 88% for PFP (Bz+)) and the jackknife classification (88% for PFP (Bz−) and 82% for PFP (Bz+)), validating that these two polymers are key contributors for detecting protein level fluctuations in serum.

We conjecture that the strong serum discrimination performance of PFP (Bz−) and PFP (Bz+) arises from their dynamic pyrene fluorescence responses, which are influenced by their high surface charge and aromaticity. At the same time, serum proteins (HSA, Fib, IgG, and Trf) differ significantly in molecular weight, surface charge, and structure. As a result, different proteins perturb the local polymer environment in distinct ways, enabling these PFPs to generate unique fluorescence response patterns. Similar behavior has been demonstrated in previously reported gold nanoparticle-based sensor arrays, where proteins with distinct surface charges and conformations produce characteristic signal patterns [23]. This differential interaction mechanism enables PFP (Bz−) and PFP (Bz+) to discriminate multiple serum proteins even in complex serum environments.

### 3.5. Development of a Multichannel Sensor Array and Sensor Optimization

**Figure 5:**
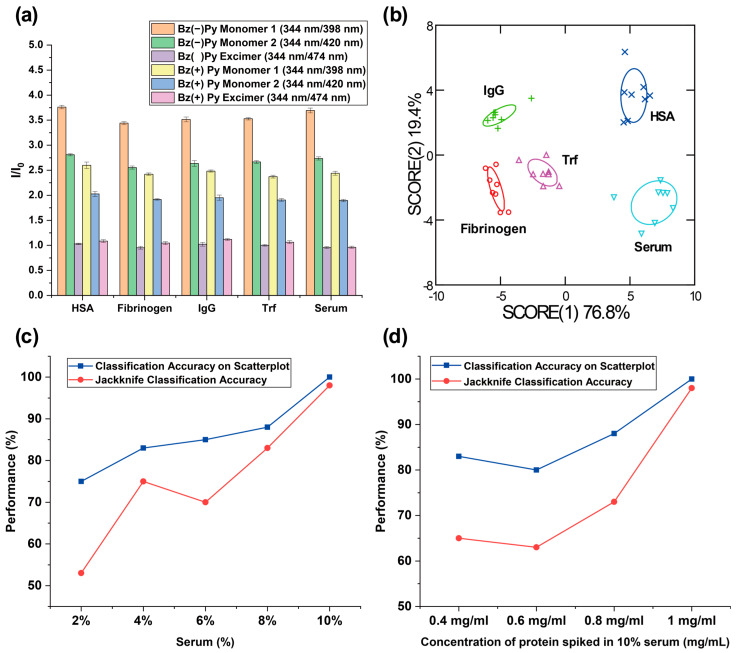
(**a**) Fluorescence bar graph obtained from 50 μg/mL PFP functionalized with Bz− and Bz+ after incubation with serum samples for 45 min (*n* = 8). (**b**) LDA plot of the fluorescence patterns generated from interactions between serum samples and PFPs functionalized with Bz− and Bz+. (**c**) Classification accuracies of discrimination analysis for varying dilutions of whole sera spiked with 10 mg/mL proteins. (**d**) Classification accuracies of discrimination analysis for sera spiked with varying concentrations of proteins.

After successful identification of polymers providing key contributions for detecting protein concentration changes in serum, we investigated the use of two polymers for profiling serum analytes to provide an even higher classification accuracy. Inspired by previously reported literature [50,51], we expected that fluorescence fingerprints formulated from multiple recognition elements would confer the ability of the sensor array to identify analytes with higher sensitivity, compared to an array-based sensing system using one type of recognition element. As expected, we were able to achieve higher classification accuracies in both the two-dimensional classification matrix (100%) and the jackknife classification (98%) by formulating fluorescence patterns with six fluorescence responses (I/I_0_) generated from PFP (Bz−) and PFP (Bz+) (Figure 5a,b). Next, we performed unknown identification to further evaluate the reproducibility of the classification accuracy achieved with the sensor array fabricated with two polymers. Out of 40 serum samples, 38 samples were classified correctly, yielding a 95% unknown classification accuracy.

We conducted sensing experiments and discrimination analyses using serum samples at varying spiked protein concentrations in sera (2–8%) to further optimize the sensitivity of the sensor array constructed from PFP (Bz−) and PFP (Bz+). Then, we compared the resulting discrimination performance with previous results obtained from 10% serum samples. As shown in Figure 5c and Figure S13, the classification accuracy of the array-based sensor gradually increased with increasing serum concentration. This trend suggests that the concentrations of both endogenous and spiked proteins at lower serum percentages are insufficient to generate distinguishable fluorescence response patterns for different serum analytes. These results indicate that the absolute concentration of serum proteins is crucial for determining sensing performance. We also prepared serum samples in which protein concentrations below 1 mg/mL were spiked into 10% serum. The discrimination results (Figure 5d) demonstrated that increasing the concentration of protein spiked into the serum corresponded to higher classification accuracy. Notably, the newly designed high-content sensor array exhibited excellent sensitivity toward HSA, successfully detecting a 0.4 mg/mL change in HSA concentration in serum relative to other proteins (Figure S14). Additionally, we examined the fluorescence responses of PFP (Bz−) and PFP (Bz+) under varying glucose concentrations to evaluate the robustness of the sensor and to confirm that the analyte discrimination performance of the polymer sensor array primarily arises from polymer–protein interactions rather than potential variations in glucose levels. As shown in Figure S15, no observable changes in the fluorescence spectra were detected, indicating stable sensor performance under physiologically relevant conditions.

These results highlight the potential of this novel sensor array as a diagnostic tool for detecting diseases associated with serum albumin fluctuations. HSA levels in whole serum of healthy adults typically range from approximately 35–50 mg/mL, while pathological conditions such as liver disease, kidney disease, or diabetic nephropathy can reduce HSA levels significantly below 35 mg/mL [29,47,48]. The sensitivity of the PFP sensor array observed with 0.4 mg/mL of HSA spiked in 10% serum is smaller than these pathological variations, indicating that the PFP sensor array is sufficiently sensitive to detect clinically relevant changes in HSA. This highlights its potential for monitoring clinically meaningful fluctuations in serum proteins [52,53].

## 4. Conclusions

The above-mentioned diseases manifest as small shifts in serum protein concentration at early stages. Thus, developing a sensitive sensor platform that can detect subtle protein composition changes in serum is crucial for early disease diagnosis. We developed a high-throughput multichannel polymer sensor array capable of detecting protein-level perturbations in serum to achieve this goal. We rationally designed and synthesized PFPs that can generate unique fluorescence patterns based on spontaneous polymer-protein interactions. Screening these polymers to evaluate their contributions to analyte discrimination suggested rational strategies for optimizing sensor sensitivity with only two of the polymers for detecting protein-level changes in serum. As demonstrated by the results, the optimized polymer sensor array reliably detected protein-level changes in protein-spiked serum samples with high classification accuracy and excellent reproducibility, as confirmed by the unknown classification experiment. We anticipate that this strategy can be used to develop and optimize sensor arrays to improve the sensitivity of serum-based sensors, facilitating earlier and more reliable disease detection. In addition, this polymer sensor array holds potential for sensitive protein detection in complex mixtures beyond serum for other applications.

In addition, linking the sensitivity of the newly developed PFP sensor array to advanced analyte models will be crucial for validating its potential in clinical settings. For instance, previous research demonstrates that employing clinically relevant and well-characterized analyte models is essential for assessing clinical applicability [54]. The development of accurate and reliable chemical nose sensing systems for diagnostic purposes can be realized by constructing well-established training sets. Herein, we have proposed a method to construct a sensitive polymer sensor array for serum analyte discrimination. Thus, a natural future direction is to expand this research by generating distinct fluorescence fingerprints using polymer-based sensor elements and training the sensor with patient serum samples representing diverse pathophysiological conditions, thereby further demonstrating its clinical applicability.

## Data Availability

The datasets for this study are available from the corresponding author upon reasonable request.

## Supplementary Materials

The following supporting information can be downloaded at https://www.mdpi.com/article/10.3390/s26082308/s1. Synthesis and characterization of polymer backbones and PFPs (Figures S1–10) and sensing data (Figures S11–13 and Tables S1–14) (PDF). Figure S1. Reaction schemes for synthesizing polymer backbones and PFP (Zwt). Figure S2. ^1^H NMR spectrum (400 MHz, CDCl3) of Polymer backbone 1 for synthesizing PFP (COO−). δ 8.35~7.89 (m, 0.5H), 6.09 (s, 1H), 5.80 (s, 1H), 5.05 (m, 1H), 4.50 (s, 1H), 3.53 (S, 2H), 3.39~3.23 (m, 2H), 2.41~2.22 (m, 2H), 1.88~1.85 (m, 2H) 1.46 (s, 9H). Figure S3. ^1^H NMR spectrum (400 MHz, CDCl_3_ + one drop of acetone) of Polymer backbone 2 for synthesizing PFP (Bz−). *δ* 9.11 (br, 1H) 8.22~7.86 (m, 4.9H), 5.89~5.71 (br, 2H), 4.45~4.34 (br, 1H), 3.80 (br, 2H), 3.29 (s, 2H), 2.71 (S, 2H),1.54 (s, 9H). Figure S4. ^1^H NMR spectrum (400 MHz, CDCl_3_) of Polymer backbone 3 for synthesizing PFP (TMA+) and PFP (Bz+). *δ* 8.22~7.86 (m, 0.3H), 6.09 (s, 1H), 5.82 (s, 1H), 5.05~5.01 (br, 1H), 4.57~4.51 (br, 1H), 3.66 (S, 2H), 3.40 (s, 2H), 2.20~.216 (m 2H). Figure S5. ^1^H NMR spectrum (400 MHz, D_2_O) of PFP (Zwt). *δ* 8.35~7.31 (m, 0.7H), 6.02~5.72 (m, 2H), 4.97~4.51 (m, 2H), 3.52~3.29 (m, 6H), 3.03 (s, 6H), 2.89 (s, 2H), 2.12~1.91 (m 4H). Figure S6. ^1^H NMR spectrum (400 MHz, D_2_O) of PFP (COO-). *δ* 7.94~7.32 (m, 0.4H), 6.02~5.46 (m, 2H), 5.05~4.87 (m, 2H), 3.41~3.00 (m, 4H), 2.08 (s, 2H), 1.70~1.62 (m 2H). Figure S7. ^1^H NMR spectrum (400 MHz, D_2_O) of PFP (Bz-). *δ* 8.21~6.92 (m, 4.3H), 5.74~5.50 (m, 2H), 5.09~4.43 (m, 2H), 3.37 (s, 2H), 2.92 (s, 2H), 2.50~2.46 (m 2H). Figure S8. ^1^H NMR spectrum (400 MHz, D_2_O) of PFP (TMA+). *δ* 8.36~7.32 (m, 0.4H), 6.06~5.86 (m, 2H), 4.98~4.59 (m, 2H), 3.53~3.30 (m, 6H), 3.05 (s, 9H), 2.03~1.71 (m 2H). Figure S9. ^1^H NMR spectrum (400 MHz, DMSO-d6) of PFP (Bz+). *δ* 8.35~7.89 (m, 0.7H), 7.52 (s, 5H), 5.98~5.77 (m, 2H), 4.91 (br, 1H), 4.54~4.45 (m, 3H), 3.44~3.24 (br, 2H), 2.95 (s, 6H) 2.05 (s. 2H). Figure S10. GPC traces of polymer backbones used for synthesizing (**a**) PFP (Bz−), (**b**) PFP (COO−), (**c**) PFP (Bz+) and (TMA+) recorded using a polystyrene standard, tetrahydrofuran as solvent. (**d**) GPC traces of PFP (Zwt) recorded using a PMMA standard, trifluoroethanol as solvent. Figure S11. Dynamic light scattering (DLS) analysis of **a)** PFP (Bz−) and **b)** PFP (Bz+) in the absence and presence of HSA at varying concentrations (*n* = 3). Intensity-weighted size distributions were reported to show changes in polymer assembly behavior upon addition of HSA, indicating perturbation of the polymer environment under sensing conditions. Figure S12. Fluorescence bar graphs and LDA plots of (**a**) PFP (Bz+) (classification accuracy on the scatterplot: 85%; Jackknife classification: 75%; *n* = 8), (**b**) PFP (COO−) (classification accuracy on the scatterplot: 75%; Jackknife classification: 65%; *n* = 8), (**c**) PFP (TMA+) (classification accuracy on the scatterplot: 65%; Jackknife classification: 53%; *n* = 8), and (**d**) PFP (Zwt) (classification accuracy on the scatterplot: 80%; Jackknife classification: 68%; *n* = 8). All classification values were used to plot the heatmap in Figure 4c, along with those obtained from PFP (Bz−). Figure S13. LDA plots generated from fluorescence responses (I/I_0_) of PFP (Bz−) and PFP (Bz+) against (**a**) 2% serum samples (classification accuracy on the scatterplot: 75%; Jackknife classification: 53%; *n* = 8), (**b**) 4% serum samples (classification accuracy on the scatterplot: 83%; Jackknife classification: 75%; *n* = 8), and (**c**) 6% serum samples (classification accuracy on the scatterplot: 85%; Jackknife classification: 70%; *n* = 8) (**d**) 8% serum samples (classification accuracy on the scatterplot: 88%; Jackknife classification: 83%; *n* = 8). Figure S14. LDA plots generated from fluorescence responses (I/I_0_) of PFP (Bz−) and PFP (Bz+) against 10% sera spiked with (**a**) 0.4 mg/mL proteins (classification accuracy on the scatterplot: 83%; Jackknife classification: 65%; *n* = 8), (**b**) 0.6 mg/mL proteins (classification accuracy on the scatterplot: 80%; Jackknife classification: 63%; *n* = 8), and (**c**) 0.8 mg/mL proteins (classification accuracy on the scatterplot: 90%; Jackknife classification: 73%; *n* = 8). Figure S15. Fluorescence responses of (**a**) PFP (Bz−) and (**b**) PFP (Bz+) under varying concentrations (*n* = 3). Table S1. Normalized fluorescence responses (I/I_0_) and LDA outputs from PFP (Bz−). Score (1) and (2) correspond to Figure 4b. Table S2. Normalized fluorescence responses (I/I_0_) and LDA outputs from PFP (Bz+). Score (1) and (2) correspond to Figure S12a. Table S3. Normalized fluorescence responses (I/I_0_) and LDA outputs from PFP (COO−). Score (1) and (2) correspond to Figure S12b. Table S4. Normalized fluorescence responses (I/I_0_) and LDA outputs from PFP (TMA+). Score (1) and (2) correspond to Figure S12c. Table S5. Normalized fluorescence responses (I/I_0_) and LDA outputs from PFP (Zwt). Score (1) and (2) correspond to Figure S12d. Table S6. LDA output for the training set of the sensor array with six fluorescence channels, constructed using fluorescence responses (I/I_0_) of PFP (Bz−) and PFP (Bz+). Scores (1) and (2) correspond to Figure 5a. Table S7. Identification of unknown protein-spiked serum samples using the training set from Figure 5b and Table S6. The overall prediction accuracy for unknown samples using the training set is 100%. Table S8. Normalized fluorescence responses (I/I_0_) and LDA outputs from the sensor array constructed with PFP (Bz−) and PFP (Bz+). Score (1) and (2) correspond to Figure S12a. Table S9. Normalized fluorescence responses (I/I_0_) and LDA outputs from the sensor array constructed with PFP (Bz−) and PFP (Bz+). Score (1) and (2) correspond to Figure S12b. Table S10. Normalized fluorescence responses (I/I_0_) and LDA outputs from the sensor array constructed with PFP (Bz−) and PFP (Bz+). Score (1) and (2) correspond to Figure S12c. Table S11. Normalized fluorescence responses (I/I_0_) and LDA outputs from the sensor array constructed with PFP (Bz−) and PFP (Bz+). Score (1) and (2) correspond to Figure S12d. Table S12. Normalized fluorescence responses (I/I_0_) and LDA outputs from the sensor array constructed with PFP (Bz−) and PFP (Bz+). Score (1) and (2) correspond to Figure S13a. Table S13. Normalized fluorescence responses (I/I_0_) and LDA outputs from the sensor array constructed with PFP (Bz−) and PFP (Bz+). Score (1) and (2) correspond to Figure S13b. Table S14. Normalized fluorescence responses (I/I_0_) and LDA outputs from the sensor array constructed with PFP (Bz−) and PFP (Bz+). Score (1) and (2) correspond to Figure S13c.
