# Supplementary material for: A Sensitive Multichannel Fluorescent Polymer Sensor Array for the Detection of Protein Fluctuations in Serum
Source: Sensors (Basel). 2026 Apr 9;26(8):2308. doi: 10.3390/s26082308 (PMC13119533; doi:10.3390/s26082308)
Supplement: Supplementary file 1 [file sensors-26-02308-s001.zip › sensors-4198060-supplementary.pdf]

A Sensitive Multichannel Fluorescent Polymer Sensor Array for the Detection of Protein Fluctuations in Serum

Junwhee Yang, Colby Alves, Kanwal Nazir, Mingdi Jiang, Nicolas Araujo and Vincent M. Rotello *

Department of Chemistry, University of Massachusetts Amherst, 710 North Pleasant Street,
Amherst, MA 01003, USA; junwheeyang@umass.edu (J.Y.); colbyalves1234@gmail.com (C.A.); knazir@umass.edu (K.N.); mingdijiang@umass.edu (M.J.); naraujo@umass.edu (N.A.)

* Correspondence: rotello@chem.umass.edu

1. *Synthesis and characterization of polymer backbones and PFPs*

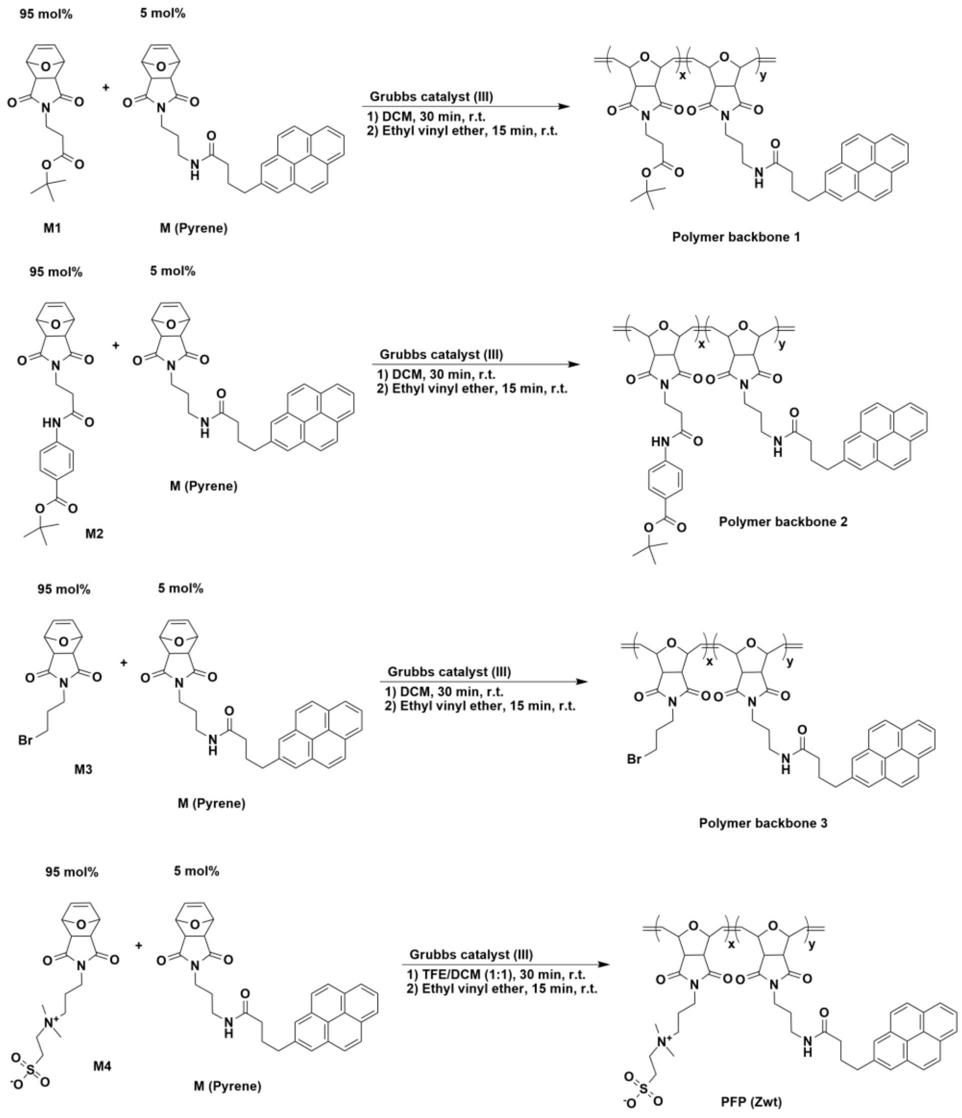

Figure S1. Reaction schemes for synthesizing polymer backbones and PFP (Zwt).

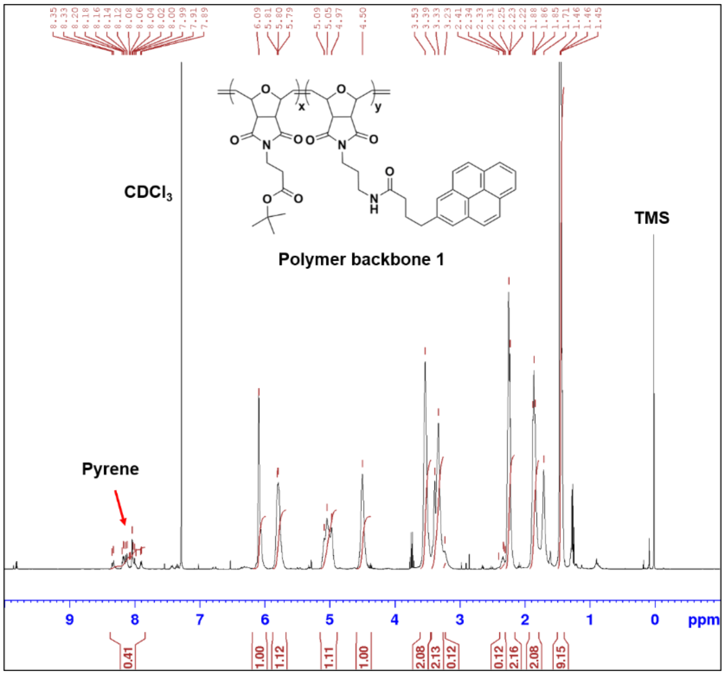

Figure S2. ^{1}H NMR spectrum (400 MHz, $CDCl_3$) of Polymer backbone 1 for synthesizing PFP (COO−). δ 8.35~7.89 (m, 0.5H), 6.09 (s, 1H), 5.80 (s, 1H), 5.05 (m, 1H), 4.50 (s, 1H), 3.53 (S, 2H), 3.39~3.23 (m, 2H), 2.41~2.22 (m, 2H), 1.88~1.85 (m, 2H) 1.46 (s, 9H)

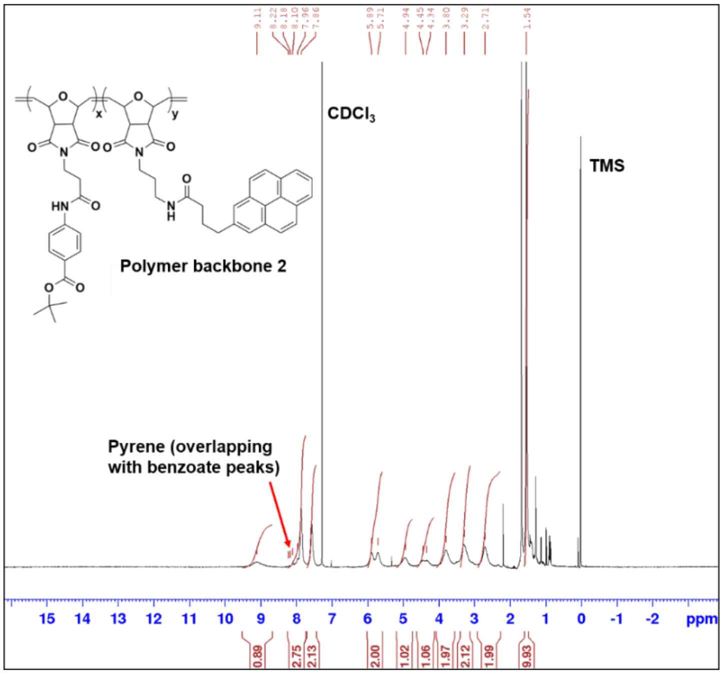

Figure S3. ^{1}H NMR spectrum (400 MHz, $CDCl_3$ + one drop of acetone) of Polymer backbone 2 for synthesizing PFP (Bz−). δ 9.11 (br, 1H) 8.22~7.86 (m, 4.9H), 5.89~5.71 (br, 2H), 4.45~4.34 (br, 1H), 3.80 (br, 2H), 3.29 (s, 2H), 2.71 (S, 2H),1.54 (s, 9H).

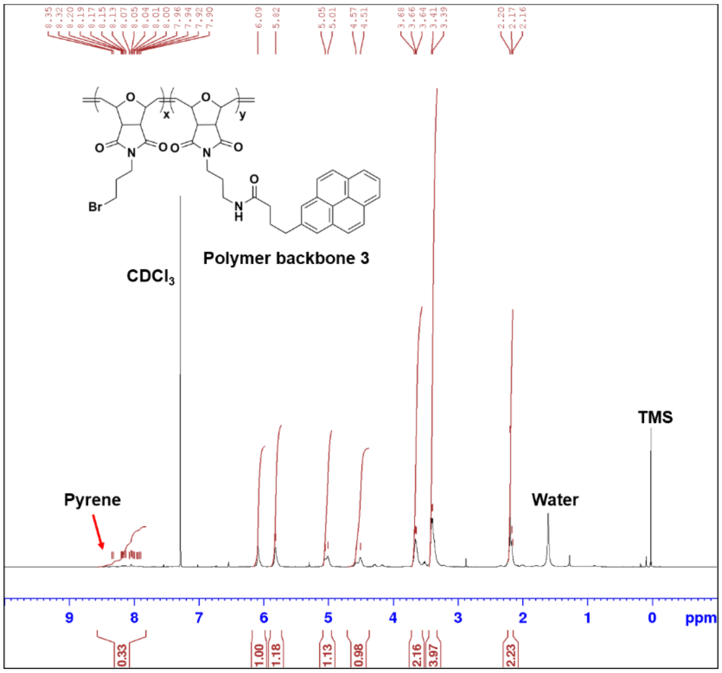

Figure S4. ^{1}H NMR spectrum (400 MHz, $CDCl_3$) of Polymer backbone 3 for synthesizing PFP (TMA+) and PFP (Bz+). δ 8.22~7.86 (m, 0.3H), 6.09 (s, 1H), 5.82 (s, 1H), 5.05~5.01 (br, 1H), 4.57~4.51 (br, 1H), 3.66 (S, 2H), 3.40 (s, 2H), 2.20~.216 (m 2H).

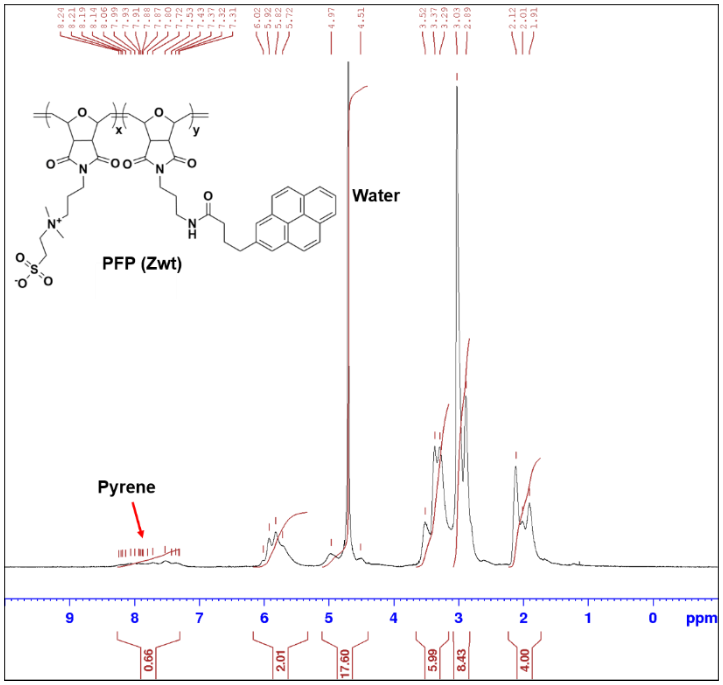

Figure S5. ^{1}H NMR spectrum (400 MHz, D_2O) of PFP (Zwt). δ 8.35~7.31 (m, 0.7H), 6.02~5.72 (m, 2H), 4.97~4.51 (m, 2H), 3.52~3.29 (m, 6H), 3.03 (s, 6H), 2.89 (s, 2H), 2.12~1.91 (m 4H).

Synthesis of PFP (COO−) and PFP (Bz−)

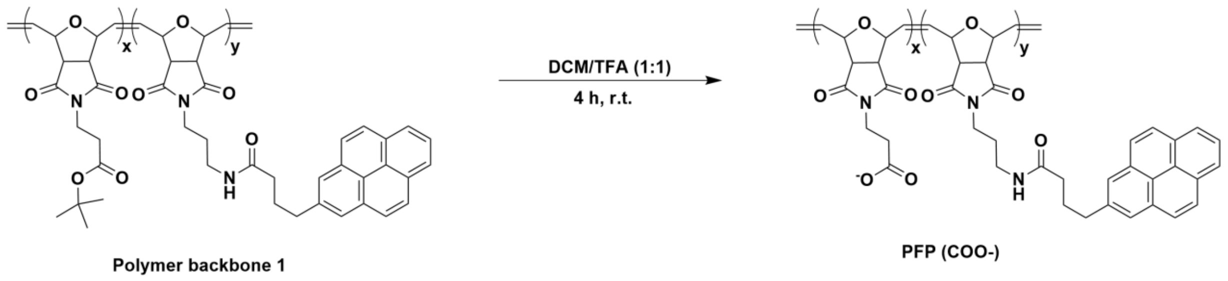

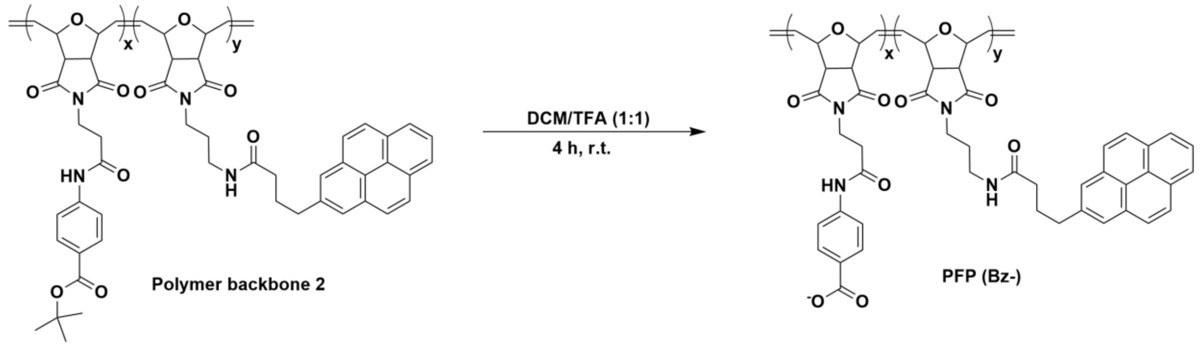

Generally, to a 20 mL glass vial, 50 mg of each polymer was added, dissolved in 3mL of DCM, and caped with rubber septum. Then, 3 mL of trifluoroacetic acid (TFA) was slowly added using a 5 mL disposable syringe. The deprotection reaction was allowed to proceed at room temperature for 4 hours. Afterwards, the crude product was air-dried. Next, 1 mL of deionized water was added, followed by the slow addition of ammonium hydroxide until the polymer became soluble in the aqueous solution. Finally, each polymer solution was dialyzed against a 5 mM phosphate buffer (pH 7.4) for three days, filtered through a 0.22 μm PES filter, and lyophilized to obtain the pure PFP (COO–) and PFP (Bz–).

Synthesis of **PFP (TMA+)**

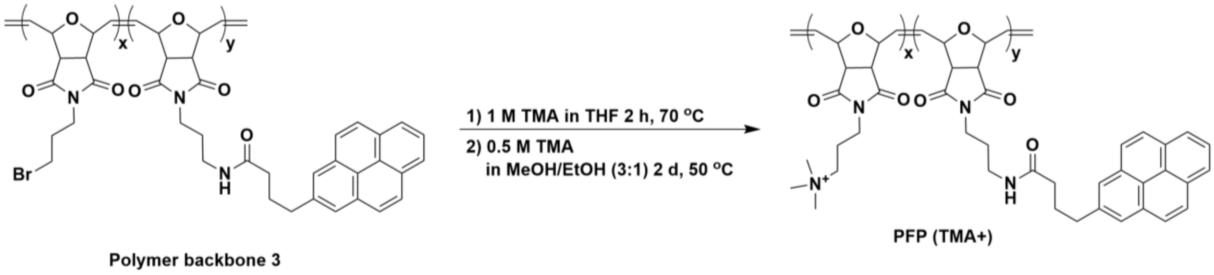

To a 20 mL glass vial, 50 mg of each polymer was added and dissolved in 1 M trimethylamine (TMA) in tetrahydrofuran (THF). The vial was then sealed tightly using Teflon tape. After 2 hours of stirring at 70 °C, the polymer started to precipitate in THF. The reaction mixture was cooled to the room temperature, air-dried, and re-dissolved in 0.5 M TMA in methanol/ethanol (3:1). The reaction mixture was allowed to stir for an additional 2 days. Afterwards, the reaction mixture was air-dried, dissolved in 2 mL of dimethyl sulfoxide (DMSO), dialyzed against deionized water for three days, and filtered through a 0.22 μm PES filter. After purification, the polymer was lyophilized to obtain pure PFP (TMA+).

Synthesis of **PFP (Bz+)**

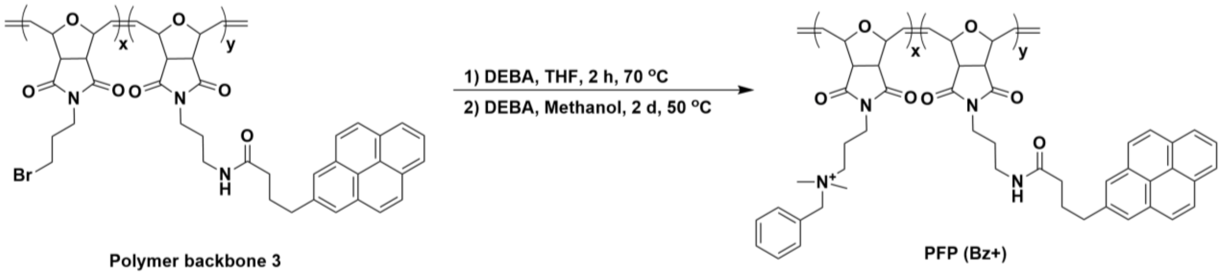

To a 20 mL glass vial, 50 mg of each polymer was added and dissolved in 5 mL of THF, and 500 μL of dimethyl ethylbenzylamine (DEBA) was added to the polymer solution. The vial was then sealed tightly using Teflon tape and stirred for 2 hours at 70 °C. After 2 hours, the polymer backbone functionalized with DEBA started to precipitate. The reaction mixture was cooled to room temperature, air-dried, and redissolved in 5 mL of methanol, followed by the addition of 500 μL of DEBA. The reaction mixture was allowed to stir for an additional 2 days. Afterwards, the reaction mixture was air-dried, dissolved in 3 mL of dimethyl sulfoxide (DMSO), dialyzed against deionized water for three days, and filtered through a 0.22 μm PES filter. After purification, the polymer was lyophilized to obtain pure **PFP (Bz+)**.

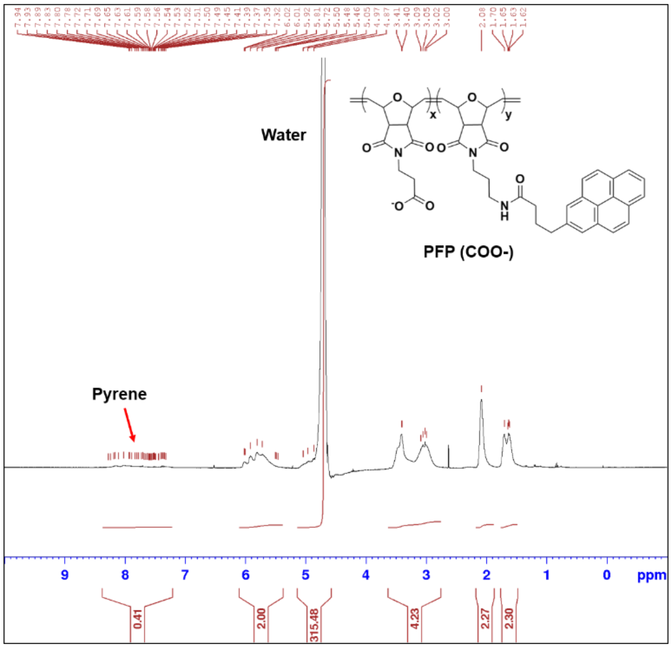

Figure S6. ^{1}H NMR spectrum (400 MHz, D_2O) of PFP (COO-). δ 7.94~7.32 (m, 0.4H), 6.02~5.46 (m, 2H), 5.05~4.87 (m, 2H), 3.41~3.00 (m, 4H), 2.08 (s, 2H), 1.70~1.62 (m 2H).

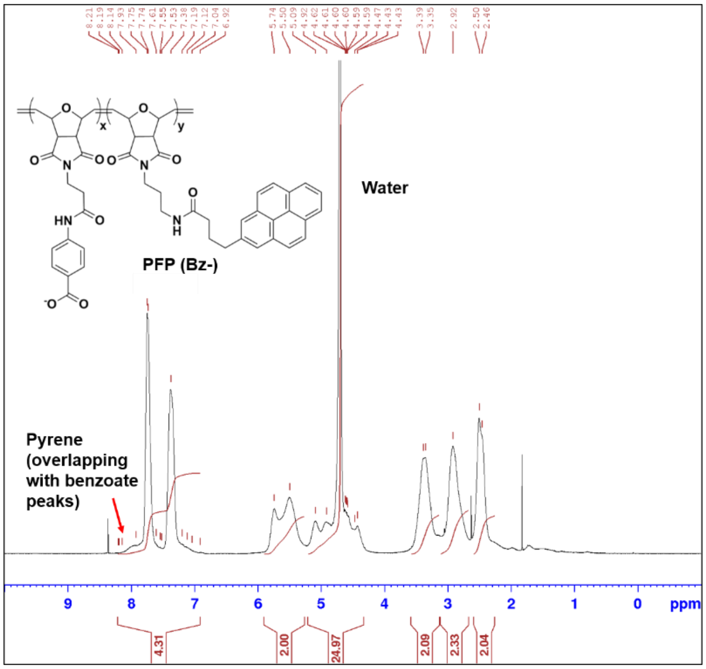

Figure S7. ^{1}H NMR spectrum (400 MHz, D_2O) of PFP (Bz-). δ 8.21~6.92 (m, 4.3H), 5.74~5.50 (m, 2H), 5.09~4.43 (m, 2H), 3.37 (s, 2H), 2.92 (s, 2H), 2.50~2.46 (m 2H).

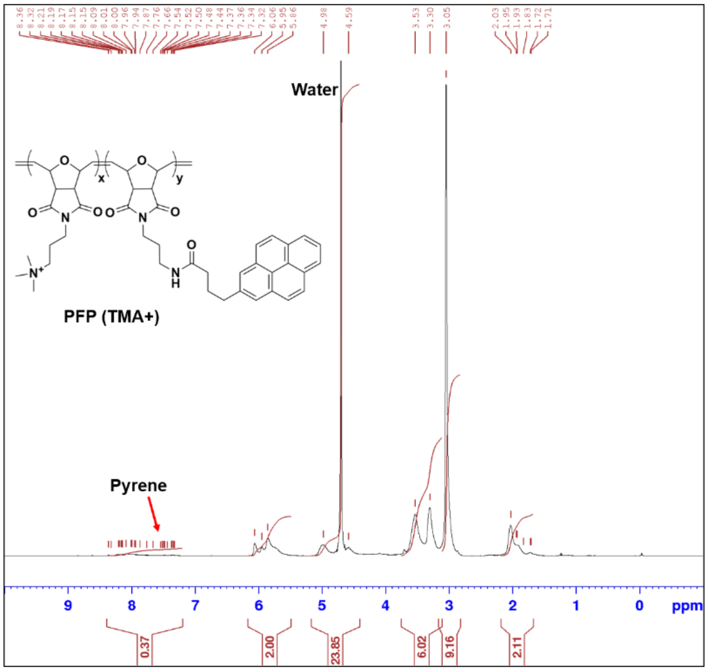

Figure S8. ^{1}H NMR spectrum (400 MHz, D_2O) of PFP (TMA+). δ 8.36~7.32 (m, 0.4H), 6.06~5.86 (m, 2H), 4.98~4.59 (m, 2H), 3.53~3.30 (m, 6H), 3.05 (s, 9H), 2.03~1.71 (m 2H).

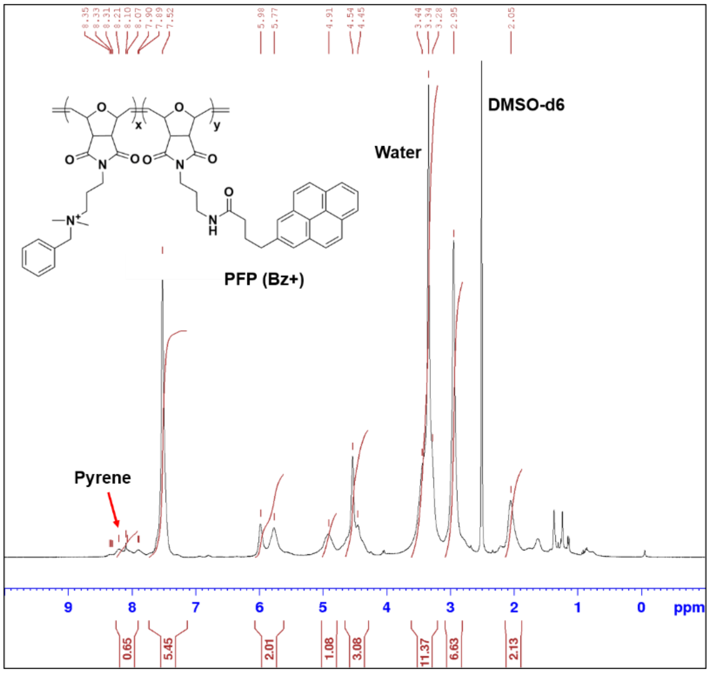

Figure S9. ^{1}H NMR spectrum (400 MHz, DMSO-d6) of PFP (Bz+). δ 8.35~7.89 (m, 0.7H), 7.52 (s, 5H), 5.98~5.77 (m, 2H), 4.91 (br, 1H), 4.54~4.45 (m, 3H), 3.44~3.24 (br, 2H), 2.95 (s, 6H) 2.05 (s. 2H).

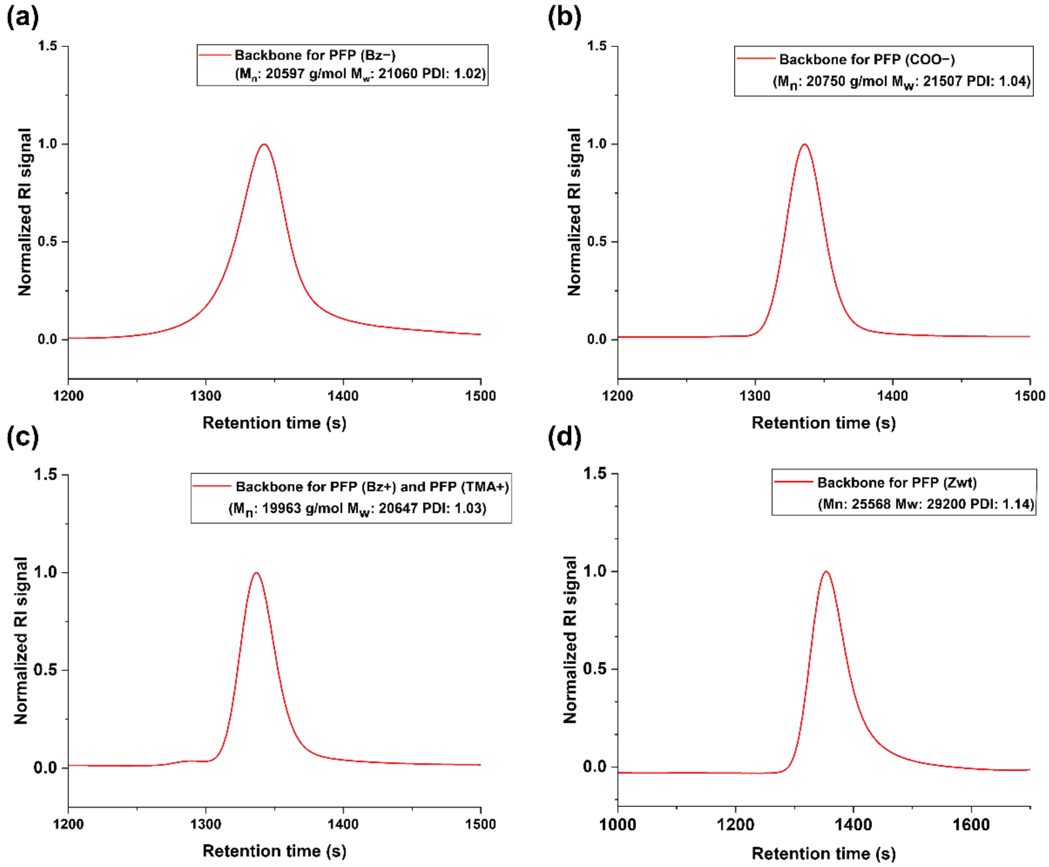

Figure S10. GPC traces of polymer backbones used for synthesizing **a)** PFP (Bz−), **b)** PFP (COO−), **c)** PFP (Bz+) and (TMA+) recorded using a polystyrene standard, tetrahydrofuran as solvent. **d)** GPC traces of PFP (Zwt) recorded using a PMMA standard, trifluoroethanol as solvent.

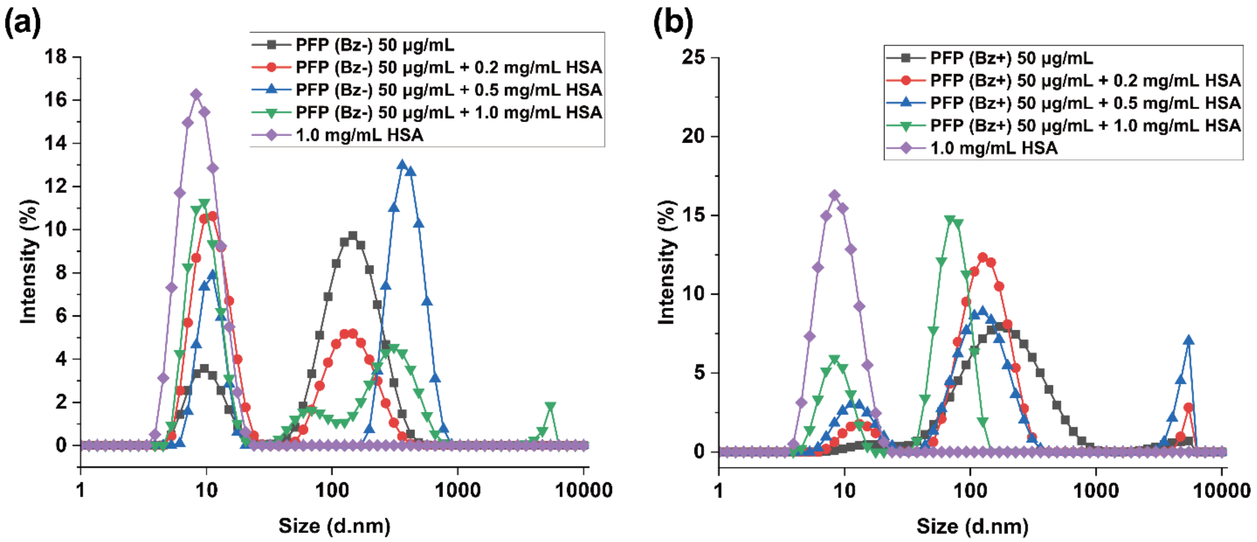

Figure S11. Dynamic light scattering (DLS) analysis of **a)** PFP (Bz−) and **b)** PFP (Bz+) in the absence and presence of HSA at varying concentrations (*n* = 3). Intensity-weighted size distributions were reported to show changes in polymer assembly behavior upon addition of HSA, indicating perturbation of the polymer environment under sensing conditions.

2. *Sensing data*

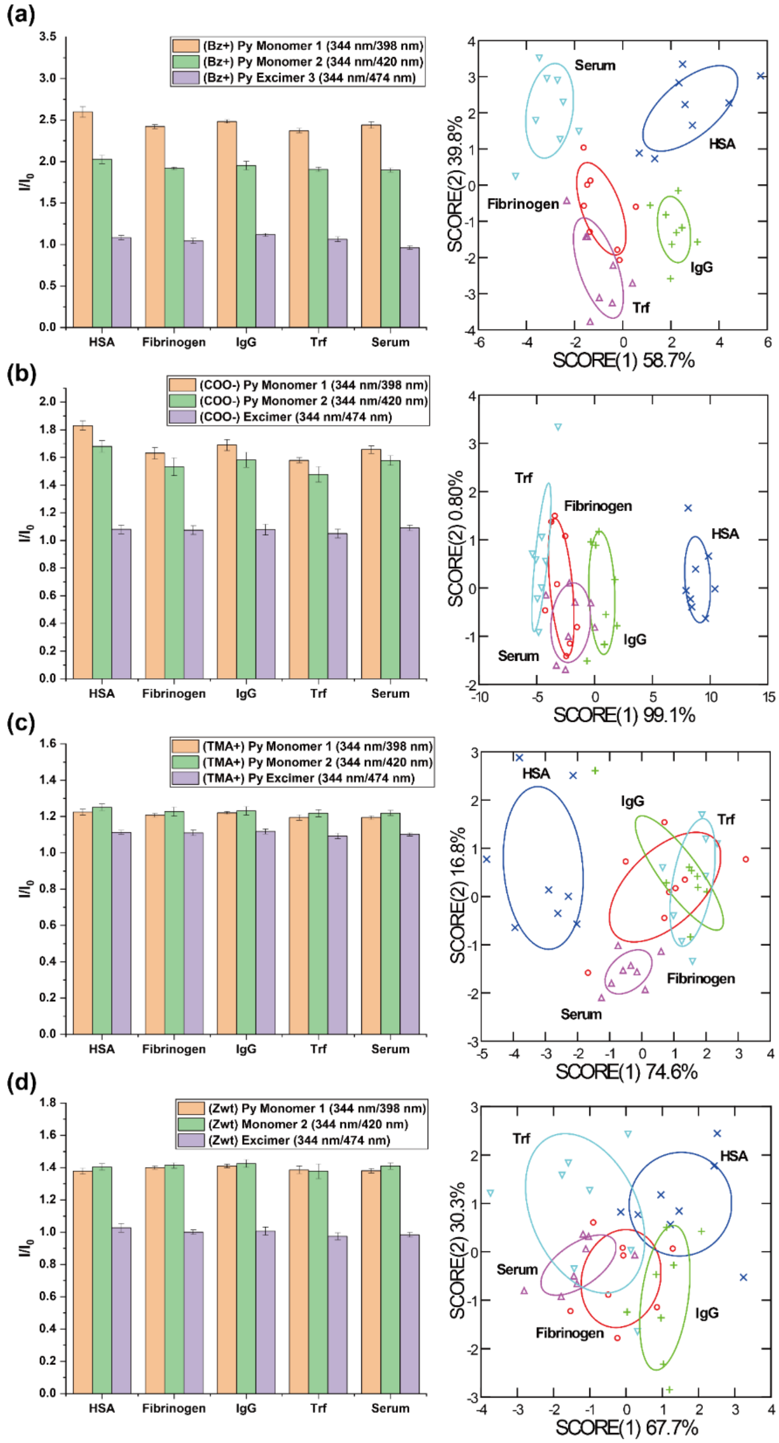

Figure S12. Fluorescence bar graphs and LDA plots of **a)** PFP (Bz+) (classification accuracy on the scatterplot: 85%; Jackknife classification: 75%; n = 8), **b)** PFP (COO−) (classification accuracy on the scatterplot: 75%; Jackknife classification: 65%; n = 8), **c)** PFP (TMA+) (classification accuracy on the scatterplot: 65%; Jackknife classification: 53%; n = 8), and **d)** PFP (Zwt) (classification accuracy on the scatterplot: 80%; Jackknife classification: 68%; n = 8). All classification values were used to plot the heatmap in **Figure 4c**, along with those obtained from PFP (Bz−).

Table S1. Normalized fluorescence responses (I/I_0) and LDA outputs from PFP (Bz−). Score (1) and (2) correspond to **Figure 4b**.

Sample name	I/I_0			LDA output	
	Py 1	Py 2	Py Ex	Score (1)	Score (2)
HSA	3.693	2.765	1.008	3.935	0.560
HSA	3.737	2.801	1.036	4.275	1.862
HSA	3.763	2.814	1.033	5.309	1.803
HSA	3.775	2.813	1.037	5.172	2.160
HSA	3.761	2.829	1.021	6.744	0.868
HSA	3.773	2.832	1.056	4.728	2.795
HSA	3.802	2.798	1.014	6.315	1.608
HSA	3.795	2.828	1.032	6.353	1.904
Fibrinogen	3.403	2.523	0.936	-5.452	-2.308
Fibrinogen	3.476	2.609	0.998	-4.799	0.190
Fibrinogen	3.445	2.558	0.960	-4.980	-1.184
Fibrinogen	3.419	2.555	0.961	-5.513	-1.388
Fibrinogen	3.418	2.543	0.933	-4.193	-2.633
Fibrinogen	3.426	2.537	0.919	-3.505	-3.125
Fibrinogen	3.453	2.521	0.934	-4.823	-1.743
Fibrinogen	3.484	2.590	0.987	-4.914	0.121
IgG	3.462	2.555	0.963	-5.123	-0.745
IgG	3.489	2.572	0.987	-5.565	0.476
IgG	3.454	2.608	0.997	-5.083	-0.098
IgG	3.591	2.735	1.075	-2.858	3.234
IgG	3.515	2.671	1.051	-5.053	2.252
IgG	3.511	2.653	1.027	-4.358	1.322
IgG	3.541	2.647	1.035	-4.788	2.231
IgG	3.560	2.647	1.044	-5.128	2.918
Trf	3.535	2.655	0.990	-1.616	-0.300
Trf	3.493	2.723	1.037	-2.117	0.290
Trf	3.501	2.648	1.011	-3.655	0.469
Trf	3.533	2.673	0.998	-1.351	-0.260
Trf	3.525	2.671	0.995	-1.294	-0.493
Trf	3.533	2.641	0.979	-1.526	-0.632
Trf	3.568	2.679	1.001	-0.886	0.243
Trf	3.541	2.635	0.992	-2.506	0.214

Serum	3.629	2.677	0.922	4.905	-3.047
Serum	3.657	2.762	0.965	6.134	-2.036
Serum	3.631	2.709	0.971	3.136	-1.090
Serum	3.717	2.764	0.971	6.603	-1.045
Serum	3.708	2.763	0.964	6.890	-1.485
Serum	3.735	2.735	0.935	7.822	-2.104
Serum	3.752	2.756	0.977	6.212	-0.115
Serum	3.718	2.721	0.942	6.549	-1.693

Table S2. Normalized fluorescence responses (I/I_0) and LDA outputs from PFP (Bz+). Score (1) and (2) correspond to **Figure S12a**.

Sample name	I/I_0			LDA output	
	Py 1	Py 2	Py Ex	Score (1)	Score (2)
HSA	2.516	1.945	1.076	1.327	0.731
HSA	2.507	1.963	1.058	0.685	0.887
HSA	2.589	2.036	1.093	2.895	1.654
HSA	2.701	2.084	1.136	5.726	3.019
HSA	2.648	2.084	1.116	4.415	2.274
HSA	2.598	2.044	1.078	2.588	2.229
HSA	2.608	2.035	1.065	2.337	2.828
HSA	2.624	2.021	1.063	2.488	3.344
Fibrinogen	2.398	1.885	1.084	-0.115	-2.085
Fibrinogen	2.409	1.937	1.074	-0.208	-1.800
Fibrinogen	2.392	1.918	1.043	-1.354	-1.306
Fibrinogen	2.459	1.926	1.077	0.563	-0.611
Fibrinogen	2.406	1.923	1.028	-1.595	-0.584
Fibrinogen	2.433	1.928	1.025	-1.325	0.114
Fibrinogen	2.424	1.905	1.025	-1.458	-0.002
Fibrinogen	2.450	1.923	1.007	-1.610	1.026
IgG	2.485	1.880	1.131	2.461	-1.177
IgG	2.511	1.897	1.113	2.307	-0.156
IgG	2.480	1.918	1.124	2.226	-1.315
IgG	2.442	1.946	1.133	1.984	-2.589
IgG	2.475	2.006	1.118	2.040	-1.633
IgG	2.505	2.020	1.141	3.106	-1.570
IgG	2.490	1.993	1.103	1.789	-0.821
IgG	2.478	1.958	1.087	1.134	-0.556

Trf	2.321	1.882	1.078	-1.355	-3.773
Trf	2.347	1.866	1.079	-0.975	-3.112
Trf	2.360	1.903	1.090	-0.437	-3.254
Trf	2.398	1.931	1.100	0.403	-2.709
Trf	2.394	1.950	1.074	-0.402	-2.220
Trf	2.385	1.921	1.040	-1.537	-1.428
Trf	2.390	1.894	1.011	-2.328	-0.424
Trf	2.385	1.908	1.043	-1.469	-1.421
Serum	2.351	1.845	0.958	-4.454	0.243
Serum	2.419	1.899	0.953	-3.614	1.790
Serum	2.430	1.897	0.983	-2.603	1.266
Serum	2.457	1.918	0.996	-1.824	1.489
Serum	2.461	1.898	0.972	-2.476	2.295
Serum	2.465	1.926	0.947	-3.151	2.947
Serum	2.471	1.905	0.934	-3.469	3.513
Serum	2.470	1.887	0.960	-2.713	2.898

Table S3. Normalized fluorescence responses (I/I_0) and LDA outputs from PFP (COO−). Score (1) and (2) correspond to **Figure S12b**.

**Sample name**	**I/I_0**			**LDA output**	
	**Py 1**	**Py 2**	**Py Ex**	**Score (1)**	**Score (2)**
HSA	1.858	1.704	1.117	8.413	-0.392
HSA	1.863	1.710	1.090	10.419	-0.020
HSA	1.861	1.730	1.104	9.613	-0.633
HSA	1.835	1.669	1.086	8.742	0.391
HSA	1.821	1.691	1.085	8.294	-0.229
HSA	1.824	1.679	1.091	7.917	-0.054
HSA	1.813	1.667	1.049	9.858	0.662
HSA	1.761	1.594	1.021	8.104	1.660
Fibrinogen	1.665	1.608	1.110	-2.415	-1.422
Fibrinogen	1.690	1.593	1.113	-1.487	-0.820
Fibrinogen	1.595	1.536	1.067	-4.246	-0.473
Fibrinogen	1.671	1.600	1.109	-2.103	-1.158
Fibrinogen	1.613	1.524	1.064	-3.213	0.069
Fibrinogen	1.637	1.487	1.067	-2.496	1.066
Fibrinogen	1.595	1.453	1.040	-3.406	1.488
Fibrinogen	1.580	1.454	1.032	-3.706	1.365

IgG	1.698	1.636	1.114	-0.658	-1.512
IgG	1.742	1.642	1.129	0.851	-1.170
IgG	1.720	1.612	1.103	0.985	-0.548
IgG	1.733	1.635	1.103	1.934	-0.783
IgG	1.667	1.521	1.063	-0.317	0.956
IgG	1.676	1.583	1.048	1.760	0.174
IgG	1.632	1.526	1.027	0.097	0.885
IgG	1.651	1.517	1.038	0.375	1.170
Trf	1.597	1.516	1.077	-4.951	-0.226
Trf	1.607	1.551	1.090	-4.848	-0.923
Trf	1.582	1.508	1.057	-4.593	0.002
Trf	1.558	1.463	1.042	-5.379	0.703
Trf	1.575	1.458	1.042	-4.575	1.052
Trf	1.591	1.487	1.055	-4.232	0.553
Trf	1.578	1.474	1.055	-5.074	0.587
Trf	1.549	1.364	0.982	-3.159	3.335
Serum	1.625	1.603	1.089	-3.312	-1.604
Serum	1.682	1.622	1.128	-2.517	-1.692
Serum	1.678	1.592	1.116	-2.266	-1.003
Serum	1.683	1.610	1.086	0.008	-0.815
Serum	1.636	1.561	1.065	-1.690	-0.293
Serum	1.676	1.581	1.081	-0.324	-0.310
Serum	1.623	1.523	1.088	-4.216	-0.144
Serum	1.647	1.537	1.079	-2.187	0.105

Table S4. Normalized fluorescence responses (I/I_0) and LDA outputs from PFP (TMA+). Score (1) and (2) correspond to **Figure S12c**.

**Sample name**	**I/I_0**			**LDA output**	
	**Py 1**	**Py 2**	**Py Ex**	**Score (1)**	**Score (2)**
HSA	1.212	1.240	1.107	0.331	0.771
HSA	1.206	1.236	1.104	-0.147	0.821
HSA	1.224	1.251	1.114	1.464	0.842
HSA	1.222	1.248	1.115	1.225	0.562
HSA	1.235	1.281	1.125	2.432	1.775
HSA	1.219	1.253	1.112	0.962	1.177
HSA	1.244	1.257	1.132	3.242	-0.527
HSA	1.232	1.241	1.096	2.517	2.446

Fibrinogen	1.193	1.214	1.107	-1.520	-1.232
Fibrinogen	1.207	1.263	1.134	-0.478	-0.893
Fibrinogen	1.199	1.239	1.106	-0.891	0.598
Fibrinogen	1.219	1.240	1.125	0.865	-1.153
Fibrinogen	1.223	1.248	1.119	1.302	0.057
Fibrinogen	1.207	1.221	1.101	-0.080	0.074
Fibrinogen	1.205	1.201	1.106	-0.227	-1.790
Fibrinogen	1.205	1.192	1.085	-0.064	-0.084
IgG	1.209	1.225	1.115	0.029	-1.244
IgG	1.223	1.225	1.130	1.201	-2.846
IgG	1.233	1.277	1.134	2.078	0.421
IgG	1.220	1.240	1.126	0.965	-1.364
IgG	1.220	1.247	1.114	1.122	0.503
IgG	1.221	1.217	1.104	1.315	-0.275
IgG	1.220	1.222	1.124	1.032	-2.321
IgG	1.215	1.201	1.095	0.833	-0.469
Trf	1.165	1.187	1.068	-3.742	1.208
Trf	1.187	1.216	1.083	-1.764	1.582
Trf	1.189	1.218	1.082	-1.591	1.836
Trf	1.207	1.235	1.090	0.055	2.430
Trf	1.211	1.247	1.117	0.135	0.028
Trf	1.197	1.231	1.096	-1.003	1.268
Trf	1.211	1.202	1.105	0.317	-1.648
Trf	1.192	1.212	1.098	-1.432	-0.352
Serum	1.178	1.199	1.093	-2.811	-0.802
Serum	1.191	1.233	1.115	-1.790	-0.922
Serum	1.197	1.228	1.104	-1.110	0.061
Serum	1.211	1.234	1.111	0.241	-0.065
Serum	1.194	1.217	1.104	-1.353	-0.657
Serum	1.196	1.211	1.092	-1.033	0.317
Serum	1.196	1.229	1.102	-1.187	0.366
Serum	1.192	1.203	1.094	-1.439	-0.498

Table S5. Normalized fluorescence responses (I/I_0) and LDA outputs from PFP (Zwt). Score (1) and (2) correspond to **Figure S12d**.

**Sample name**	**I/I_0**			**LDA output**	
	**Py 1**	**Py 2**	**Py Ex**	**Score (1)**	**Score (2)**

HSA	1.361	1.387	1.032	-4.834	0.772
HSA	1.363	1.400	1.015	-3.950	-0.645
HSA	1.397	1.418	1.070	-3.813	2.879
HSA	1.378	1.408	1.022	-2.900	0.137
HSA	1.373	1.403	1.011	-2.617	-0.350
HSA	1.379	1.410	1.008	-2.021	-0.571
HSA	1.414	1.438	1.065	-2.139	2.509
HSA	1.361	1.372	0.996	-2.288	0.003
Fibrinogen	1.385	1.432	1.005	-1.662	-1.590
Fibrinogen	1.403	1.402	1.011	0.720	1.530
Fibrinogen	1.404	1.420	1.002	0.865	0.082
Fibrinogen	1.404	1.430	1.001	0.717	-0.454
Fibrinogen	1.403	1.416	0.999	1.065	0.162
Fibrinogen	1.402	1.422	1.021	-0.487	0.717
Fibrinogen	1.413	1.407	0.985	3.256	0.762
Fibrinogen	1.387	1.381	0.979	1.357	0.339
IgG	1.416	1.433	1.059	-1.454	2.608
IgG	1.422	1.454	1.024	0.765	0.282
IgG	1.417	1.452	1.003	1.519	-0.838
IgG	1.423	1.444	1.016	1.552	0.536
IgG	1.401	1.405	0.985	2.029	0.097
IgG	1.405	1.411	0.998	1.475	0.606
IgG	1.403	1.407	0.992	1.729	0.417
IgG	1.395	1.394	0.982	1.748	0.189
Trf	1.349	1.314	0.948	0.651	0.598
Trf	1.387	1.400	0.967	1.581	-1.346
Trf	1.433	1.448	1.020	2.350	1.093
Trf	1.386	1.397	0.972	1.247	-0.932
Trf	1.395	1.388	0.979	1.986	0.421
Trf	1.388	1.396	0.981	0.987	-0.399
Trf	1.389	1.367	0.977	1.999	1.192
Trf	1.364	1.316	0.955	1.876	1.692
Serum	1.374	1.400	0.980	-0.583	-1.530
Serum	1.393	1.430	1.005	-0.738	-1.017
Serum	1.383	1.423	0.992	-0.946	-1.799
Serum	1.391	1.429	0.996	-0.341	-1.431
Serum	1.399	1.431	0.994	0.602	-1.136
Serum	1.362	1.392	0.972	-1.256	-2.098

Serum	1.372	1.393	0.972	-0.150	-1.561
Serum	1.367	1.385	0.961	0.105	-1.930

Table S6. LDA output for the training set of the sensor array with six fluorescence channels, constructed using fluorescence responses (I/I_0) of PFP (Bz−) and PFP (Bz+). Scores (1) and (2) correspond to **Figure 5a**.

Sample name	LDA output	
	Score (1)	Score (2)
HSA	4.513	2.013
HSA	4.827	2.104
HSA	5.124	3.723
HSA	4.608	6.353
HSA	6.051	4.185
HSA	4.571	3.864
HSA	6.163	3.441
HSA	6.541	3.660
Fibrinogen	-5.800	-1.569
Fibrinogen	-5.313	-0.600
Fibrinogen	-5.577	-2.339
Fibrinogen	-6.125	-0.836
Fibrinogen	-4.982	-3.570
Fibrinogen	-4.344	-3.543
Fibrinogen	-5.345	-2.428
Fibrinogen	-5.251	-1.837
IgG	-4.924	2.184
IgG	-5.413	2.641
IgG	-5.201	1.642
IgG	-2.610	3.494
IgG	-5.979	2.130
IgG	-5.517	2.615
IgG	-5.571	2.298
IgG	-5.406	2.484
Trf	-1.286	-1.176
Trf	-1.282	-1.027
Trf	-3.576	-0.303
Trf	-1.460	0.008
Trf	-1.711	-1.157
Trf	-1.698	-1.917
Trf	-0.456	-1.906

Trf	-2.479	-1.177
Serum	5.890	-4.845
Serum	6.923	-4.187
Serum	3.763	-2.605
Serum	7.361	-1.557
Serum	7.885	-2.353
Serum	8.331	-3.273
Serum	7.200	-2.303
Serum	7.555	-2.332

Table S7. Identification of unknown protein-spiked serum samples using the training set from **Figure 5b** and **Table S6**. The overall prediction accuracy for unknown samples using the training set is 100%.

Unknown sample #	PFP (Bz−) I/I_0			PFP (Bz+) I/I_0			True ID	Identified as
	Py 1	Py 2	Py Ex	Py 1	Py 2	Py Ex		
1	2.504	1.960	1.054	3.705	2.815	1.027	HSA	HSA
2	2.613	2.011	1.112	3.720	2.777	1.020	HSA	HSA
3	2.630	2.038	1.100	3.735	2.774	1.005	HSA	HSA
4	2.686	2.090	1.142	3.799	2.825	1.039	HSA	HSA
5	2.709	2.050	1.132	3.723	2.765	0.984	HSA	HSA
6	2.603	1.997	1.096	3.731	2.765	0.996	HSA	HSA
7	2.638	1.983	1.066	3.765	2.743	0.968	HSA	HSA
8	2.658	2.015	1.086	3.790	2.786	0.985	HSA	HSA
9	2.374	1.912	1.062	3.446	2.607	0.987	Fibrinogen	Fibrinogen
10	2.374	1.871	1.052	3.465	2.604	1.008	Fibrinogen	Fibrinogen
11	2.441	1.903	1.099	3.412	2.534	0.952	Fibrinogen	Fibrinogen
12	2.478	1.988	1.104	3.418	2.589	0.981	Fibrinogen	Fibrinogen
13	2.452	1.881	1.064	3.377	2.491	0.937	Fibrinogen	Fibrinogen
14	2.513	1.915	1.103	3.348	2.497	0.902	Fibrinogen	Fibrinogen
15	2.572	1.946	1.086	3.393	2.471	0.880	Fibrinogen	Fibrinogen
16	2.502	1.918	1.076	3.440	2.526	0.932	Fibrinogen	Fibrinogen
17	2.336	1.873	1.078	3.556	2.702	1.064	IgG	Trf
18	2.404	1.925	1.138	3.507	2.687	1.063	IgG	IgG
19	2.443	1.913	1.118	3.515	2.673	1.064	IgG	IgG
20	2.447	1.949	1.120	3.499	2.644	1.048	IgG	IgG
21	2.451	1.895	1.122	3.446	2.643	1.031	IgG	IgG
22	2.425	1.940	1.126	3.492	2.688	1.088	IgG	IgG

23	2.441	1.954	1.129	3.590	2.761	1.098	IgG	IgG
24	2.431	1.950	1.111	3.562	2.703	1.074	IgG	IgG
25	2.300	1.873	1.071	3.501	2.688	1.039	Trf	Trf
26	2.364	1.890	1.099	3.492	2.608	0.979	Trf	Trf
27	2.352	1.902	1.082	3.446	2.583	0.969	Trf	Fibrinogen
28	2.366	1.883	1.066	3.525	2.666	1.000	Trf	Trf
29	2.409	1.916	1.116	3.502	2.623	0.981	Trf	Trf
30	2.372	1.815	1.074	3.522	2.579	0.923	Trf	Trf
31	2.416	1.873	1.085	3.555	2.640	0.956	Trf	Trf
32	2.323	1.802	1.013	3.527	2.616	0.964	Trf	Trf
33	2.412	1.880	0.972	3.688	2.750	0.969	Serum	Serum
34	2.425	1.832	0.971	3.656	2.720	0.952	Serum	Serum
35	2.434	1.882	0.964	3.633	2.668	0.951	Serum	Serum
36	2.478	1.906	0.984	3.798	2.809	0.983	Serum	Serum
37	2.481	1.869	0.969	3.739	2.690	0.913	Serum	Serum
38	2.484	1.853	0.974	3.684	2.694	0.921	Serum	Serum
39	2.428	1.797	0.924	3.623	2.659	0.896	Serum	Serum
40	2.462	1.825	0.941	3.656	2.635	0.895	Serum	Serum

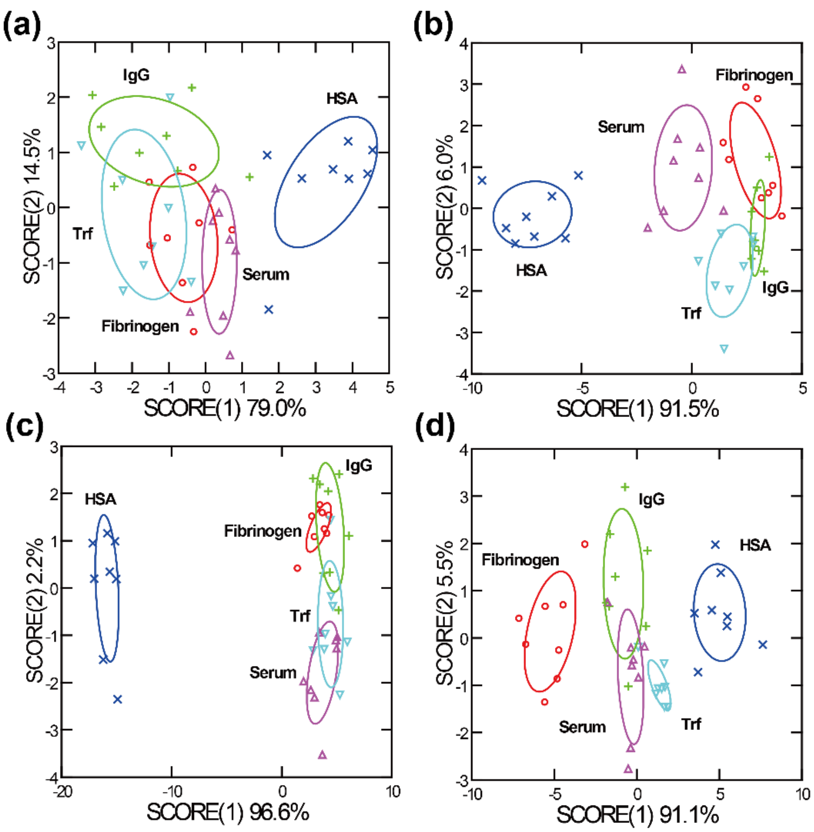

Figure S13. LDA plots generated from fluorescence responses (I/I_0) of PFP (Bz−) and PFP (Bz+) against **a)** 2% serum samples (classification accuracy on the scatterplot: 75%; Jackknife classification: 53%; *n* = 8), **b)** 4% serum samples (classification accuracy on the scatterplot: 83%; Jackknife classification: 75%; *n* = 8), and **c)** 6% serum samples (classification accuracy on the scatterplot: 85%; Jackknife classification: 70%; *n* = 8) **d)** 8% serum samples (classification accuracy on the scatterplot: 88%; Jackknife classification: 83%; *n* = 8).

Table S8. Normalized fluorescence responses (I/I_0) and LDA outputs from the sensor array constructed with PFP (Bz−) and PFP (Bz+). Score (1) and (2) correspond to **Figure S12a**.

Sample Name	PFP (Bz-)			PFP (Bz+)			LDA output	
	I/I_0			I/I_0			Score (1)	Score (2)
	Py 1	Py 2	Py Ex	Py 1	Py 2	Py Ex		
Serum	2.612	2.129	0.993	2.029	1.598	1.069	0.673	-0.570
Serum	2.649	2.109	1.000	1.978	1.586	1.055	0.218	-0.224
Serum	2.680	2.149	0.960	1.875	1.491	0.970	0.358	-0.082
Serum	2.539	2.071	0.982	2.082	1.688	1.084	0.486	-1.965
Serum	2.646	2.070	0.957	1.870	1.478	0.949	0.267	0.323
Serum	2.595	2.063	0.958	1.971	1.620	1.068	-0.423	-1.892
Serum	2.526	1.998	0.906	2.046	1.627	1.058	0.674	-2.675
Serum	2.653	2.089	0.927	1.923	1.512	0.985	0.831	-0.785
Fibrinogen	2.649	2.138	1.018	1.884	1.523	0.985	-0.349	0.707
Fibrinogen	2.578	2.113	1.011	1.843	1.494	0.997	-1.530	0.447
Fibrinogen	2.507	2.016	0.993	1.933	1.574	1.051	-1.505	-0.698
Fibrinogen	2.585	2.062	1.003	1.957	1.596	1.010	-0.174	-0.271
Fibrinogen	2.488	2.010	0.986	1.998	1.638	1.038	-0.624	-1.381
Fibrinogen	2.494	1.971	0.946	1.877	1.505	0.966	-1.025	-0.549
Fibrinogen	2.649	2.087	0.948	1.955	1.523	1.021	0.754	-0.406
Fibrinogen	2.598	2.033	0.948	2.015	1.643	1.115	-0.311	-2.265
HSA	3.009	2.363	1.036	2.025	1.604	1.022	3.899	1.201
HSA	3.044	2.395	1.004	2.022	1.593	1.015	4.430	0.607
HSA	2.684	2.087	0.984	1.963	1.526	0.957	1.686	0.959
HSA	2.979	2.352	1.033	2.021	1.624	1.027	3.466	0.698

HSA	2.780	2.191	0.993	2.074	1.721	1.101	1.728	-1.849
HSA	2.806	2.206	1.006	2.031	1.595	1.021	2.627	0.536
HSA	2.829	2.190	0.910	1.923	1.459	0.865	3.915	0.507
HSA	2.912	2.237	0.969	1.997	1.546	0.910	4.543	1.040
Trf	2.649	2.097	1.028	1.912	1.592	1.047	-1.025	-0.025
Trf	2.680	2.100	0.974	1.851	1.528	1.062	-1.456	-0.706
Trf	2.517	2.669	1.019	1.733	1.398	0.961	-2.242	-1.514
Trf	2.514	2.001	1.021	1.848	1.525	1.002	-2.278	0.496
Trf	2.555	2.044	1.027	1.853	1.453	0.952	-0.953	1.993
Trf	2.510	1.960	0.956	1.682	1.335	0.951	-3.378	1.117
Trf	2.488	1.971	0.935	1.951	1.560	0.999	-0.387	-1.341
Trf	2.379	1.846	0.892	1.851	1.452	0.949	-1.682	-1.042
IgG	2.590	2.034	0.989	1.831	1.436	0.970	-1.058	1.307
IgG	2.446	1.892	1.000	1.756	1.381	0.951	-3.073	2.047
IgG	2.534	1.973	1.012	1.864	1.499	1.007	-1.806	1.009
IgG	2.805	2.212	1.020	1.954	1.575	1.032	1.199	0.560
IgG	2.568	2.024	1.009	1.907	1.532	0.996	-0.771	0.667
IgG	2.608	2.039	0.986	1.792	1.388	0.887	-0.372	2.177
IgG	2.370	1.856	0.941	1.814	1.412	0.952	-2.485	0.382
IgG	2.419	1.918	0.974	1.742	1.367	0.916	-2.845	1.460

Table S9. Normalized fluorescence responses (I/I_0) and LDA outputs from the sensor array constructed with PFP (Bz−) and PFP (Bz+). Score (1) and (2) correspond to **Figure S12b**.

Sample Name	PFP (Bz-)			PFP (Bz+)			LDA output	
	I/I_0			I/I_0			Score (1)	Score (2)
	Py 1	Py 2	Py Ex	Py 1	Py 2	Py Ex		
Serum	3.201	2.469	1.028	2.316	1.758	1.007	-1.994	-0.470
Serum	3.155	2.442	1.028	2.316	1.758	1.016	-1.276	-0.051
Serum	3.140	2.414	1.000	2.256	1.784	1.057	0.328	0.768

Serum	3.075	2.399	1.030	2.355	1.866	1.077	-0.806	1.179
Serum	3.131	2.408	1.049	2.200	1.724	1.003	1.435	-0.037
Serum	3.140	2.396	1.014	2.354	1.839	1.075	-0.651	1.675
Serum	3.231	2.468	1.047	2.387	1.884	1.129	-0.457	3.361
Serum	3.142	2.382	0.997	2.288	1.815	1.071	0.349	1.468
Fibrinogen	3.070	2.485	1.122	2.152	1.667	1.014	3.487	0.337
Fibrinogen	2.879	2.240	0.984	2.138	1.668	0.995	4.128	-0.179
Fibrinogen	3.019	2.406	1.060	2.301	1.808	1.113	2.458	2.901
Fibrinogen	3.096	2.486	1.102	2.142	1.683	1.021	3.167	0.229
Fibrinogen	3.026	2.424	1.126	2.281	1.823	1.093	2.982	2.635
Fibrinogen	3.025	2.350	1.069	2.183	1.635	0.980	3.680	0.537
Fibrinogen	3.005	2.312	0.995	2.294	1.792	1.064	1.429	1.569
Fibrinogen	3.157	2.407	1.033	2.223	1.752	1.043	1.690	1.162
HSA	3.471	2.595	1.025	2.619	2.005	1.081	-9.506	0.670
HSA	3.322	2.570	0.989	2.511	1.908	1.054	-7.983	-0.867
HSA	3.610	2.714	1.033	2.438	1.853	1.048	-7.486	-0.200
HSA	3.423	2.634	1.065	2.431	1.896	1.081	-5.125	0.800
HSA	3.791	2.893	1.145	2.428	1.881	1.044	-8.422	-0.491
HSA	3.512	2.762	1.119	2.447	1.864	1.041	-7.123	-0.696
HSA	3.447	2.659	1.099	2.494	1.840	1.032	-6.320	0.296
HSA	3.321	2.509	1.020	2.444	1.839	1.007	-5.728	-0.730
Trf	3.086	2.427	1.060	2.121	1.626	0.956	2.345	-1.404
Trf	3.048	2.401	1.023	2.123	1.638	0.956	1.728	-1.966
Trf	3.044	2.420	1.079	2.134	1.666	0.979	2.795	-0.867
Trf	3.258	2.562	1.130	2.184	1.667	0.958	0.277	-1.302
Trf	3.117	2.479	1.086	2.182	1.717	1.004	1.358	-0.592
Trf	3.201	2.486	1.038	2.120	1.594	0.943	1.089	-1.865
Trf	2.748	2.078	0.854	2.192	1.701	0.998	2.775	-0.678
Trf	2.921	2.274	0.947	2.112	1.579	0.903	1.469	-3.407
IgG	2.958	2.267	0.998	2.211	1.688	1.001	2.966	0.501
IgG	3.104	2.384	1.028	2.152	1.646	0.986	2.699	-0.081
IgG	3.088	2.381	1.019	2.093	1.655	0.972	2.699	-1.213
IgG	3.017	2.370	1.031	2.191	1.682	1.038	3.489	1.237
IgG	2.711	2.164	0.921	2.196	1.703	1.006	2.822	-0.925
IgG	2.849	2.237	0.967	2.190	1.630	0.971	2.876	-0.769
IgG	2.971	2.346	0.965	2.101	1.633	0.998	3.055	-1.028
IgG	2.993	2.332	0.955	2.070	1.585	0.967	3.302	-1.508

Table S10. Normalized fluorescence responses (I/I_0) and LDA outputs from the sensor array constructed with PFP (Bz−) and PFP (Bz+). Score (1) and (2) correspond to **Figure S12c**.

Sample Name	PFP (Bz-)			PFP (Bz+)			LDA output	
	I/I_0			I/I_0			Score (1)	Score (2)
	Py 1	Py 2	Py Ex	Py 1	Py 2	Py Ex		
Serum	2.648	2.490	0.805	2.246	1.904	0.943	4.922	-1.097
Serum	2.764	2.645	0.859	2.402	2.017	1.022	2.656	-2.160
Serum	2.744	2.599	0.833	2.318	1.936	1.019	3.404	-0.933
Serum	2.767	2.630	0.827	2.357	1.966	1.054	1.976	-1.976
Serum	2.628	2.508	0.798	2.339	1.935	1.057	3.712	-3.518
Serum	2.638	2.496	0.794	2.198	1.787	1.068	4.951	-1.283
Serum	2.665	2.528	0.814	2.219	1.810	1.072	5.060	-0.992
Serum	2.705	2.574	0.824	2.407	2.009	1.007	3.002	-2.286
Fibrinogen	2.853	2.690	0.867	2.287	1.930	1.003	2.939	1.020
Fibrinogen	2.791	2.621	0.845	2.422	2.011	1.008	3.905	1.243
Fibrinogen	2.735	2.542	0.796	2.442	2.016	1.031	4.128	1.199
Fibrinogen	2.865	2.697	0.862	2.487	2.055	1.058	2.750	1.519
Fibrinogen	2.799	2.637	0.864	2.362	1.966	0.981	4.272	1.508
Fibrinogen	2.816	2.681	0.883	2.369	1.910	0.974	3.699	1.594
Fibrinogen	2.837	2.678	0.837	2.334	1.923	0.984	1.451	0.411
Fibrinogen	2.800	2.642	0.850	2.297	1.864	0.962	3.451	1.718
HSA	3.374	3.260	0.899	2.705	2.194	0.949	-14.854	-2.320
HSA	3.581	3.427	0.932	2.900	2.342	0.977	-17.116	0.932
HSA	3.490	3.383	0.943	2.817	2.278	0.978	-16.113	-1.497
HSA	3.490	3.334	0.910	2.963	2.396	0.991	-15.577	0.325
HSA	3.481	3.317	0.911	2.953	2.394	0.941	-15.086	0.970
HSA	3.534	3.384	0.918	3.056	2.459	0.981	-16.970	0.226

HSA	3.523	3.363	0.926	3.075	2.483	0.958	-15.794	1.134
HSA	3.426	3.283	0.899	3.034	2.401	0.967	-15.013	0.152
Trf	2.642	2.514	0.843	2.244	1.880	0.955	5.998	-1.084
Trf	2.683	2.528	0.818	2.385	1.978	0.977	4.614	-0.395
Trf	2.600	2.464	0.801	2.378	1.975	1.012	5.252	-2.288
Trf	2.750	2.625	0.872	2.424	1.988	1.018	4.451	-0.214
Trf	2.744	2.649	0.889	2.329	1.909	0.966	3.827	-1.337
Trf	2.729	2.558	0.821	2.433	1.977	0.971	4.461	1.494
Trf	2.701	2.567	0.811	2.416	1.950	1.003	2.803	-1.353
Trf	2.715	2.594	0.856	2.351	1.939	0.942	3.898	-1.041
IgG	2.704	2.549	0.845	2.225	1.840	0.984	6.068	1.123
IgG	2.798	2.627	0.867	2.348	1.950	0.995	5.182	2.393
IgG	2.725	2.557	0.820	2.454	2.031	1.019	4.307	0.311
IgG	2.673	2.503	0.803	2.499	2.081	1.066	5.140	-0.448
IgG	2.829	2.645	0.834	2.398	1.962	0.966	2.834	2.349
IgG	2.786	2.589	0.811	2.290	1.896	0.946	3.463	2.233
IgG	2.754	2.545	0.802	2.407	2.019	0.953	4.156	2.018
IgG	2.687	2.538	0.806	2.351	1.872	0.946	3.792	0.347

Table S11. Normalized fluorescence responses (I/I_0) and LDA outputs from the sensor array constructed with PFP (Bz−) and PFP (Bz+). Score (1) and (2) correspond to **Figure S12d**.

**Sample Name**	**PFP (Bz-)**			**PFP (Bz+)**			**LDA output**	
	**I/I_0**			**I/I_0**			**Score (1)**	**Score (2)**
	**Py 1**	**Py 2**	**Py Ex**	**Py 1**	**Py 2**	**Py Ex**		
Serum	3.392	2.571	1.006	2.915	2.272	1.169	-0.344	-0.202
Serum	3.360	2.528	0.975	2.731	2.116	1.083	0.104	-0.832
Serum	3.432	2.661	1.035	2.807	2.147	1.102	-0.204	-0.458
Serum	3.326	2.599	1.052	2.954	2.290	1.180	-1.768	0.759
Serum	3.572	2.762	1.075	2.759	2.128	1.107	-0.294	-0.573
Serum	3.153	2.378	0.897	2.762	2.179	1.125	-0.512	-2.762

Serum	3.684	2.820	1.102	2.846	2.232	1.156	0.473	-0.177
Serum	3.419	2.587	0.993	2.510	1.998	1.044	-0.365	-2.319
Fibrinogen	3.283	2.601	1.113	2.506	2.004	1.113	-7.102	0.408
Fibrinogen	3.313	2.641	1.079	2.534	1.980	1.076	-4.781	-0.871
Fibrinogen	3.189	2.536	1.079	2.687	2.171	1.190	-6.691	-0.144
Fibrinogen	3.237	2.530	1.068	2.594	2.046	1.106	-5.512	0.660
Fibrinogen	3.364	2.614	1.052	3.436	2.599	1.367	-3.115	1.974
Fibrinogen	3.344	2.616	1.088	2.612	2.041	1.095	-4.434	0.692
Fibrinogen	3.139	2.370	0.967	2.668	2.155	1.183	-5.544	-1.366
Fibrinogen	3.374	2.622	1.078	2.667	2.119	1.154	-4.687	-0.265
HSA	3.325	2.510	0.926	2.940	2.209	1.068	3.717	-0.721
HSA	3.758	2.809	1.071	3.147	2.379	1.157	4.764	1.973
HSA	3.958	3.017	1.138	3.204	2.417	1.190	5.094	1.381
HSA	3.770	2.871	1.075	3.091	2.368	1.149	5.466	0.254
HSA	3.647	2.801	1.050	3.256	2.442	1.210	3.502	0.523
HSA	3.691	2.815	1.059	3.195	2.431	1.189	4.549	0.589
HSA	3.659	2.769	1.035	3.178	2.433	1.173	5.493	0.449
HSA	3.671	2.769	1.010	3.136	2.394	1.122	7.634	-0.141
Trf	3.428	2.615	1.020	2.845	2.201	1.125	0.093	-0.193
Trf	3.465	2.667	1.015	2.873	2.182	1.094	1.661	-0.541
Trf	3.432	2.627	1.006	2.787	2.164	1.092	1.288	-1.065
Trf	3.352	2.570	0.990	2.832	2.224	1.111	1.513	-1.072
Trf	3.447	2.612	0.994	2.824	2.198	1.106	1.710	-1.038
Trf	3.288	2.446	0.916	2.909	2.234	1.129	1.161	-1.183
Trf	3.074	2.360	0.899	2.629	2.015	0.976	1.680	-1.457
Trf	3.259	2.551	0.979	2.681	2.073	1.015	1.873	-1.456
IgG	3.472	2.677	1.105	2.874	2.232	1.148	-1.607	2.196
IgG	3.519	2.701	1.080	2.894	2.205	1.146	-1.294	1.299
IgG	3.519	2.240	0.882	2.837	2.203	1.163	-0.699	3.196
IgG	3.299	2.524	0.977	2.874	2.232	1.148	-0.512	-1.025
IgG	3.347	2.546	1.010	2.894	2.205	1.146	-1.678	0.671
IgG	3.539	2.686	1.075	2.837	2.203	1.163	-1.852	0.744
IgG	3.395	2.625	1.036	2.849	2.206	1.108	0.569	0.246
IgG	3.572	2.714	1.076	2.791	2.097	1.057	0.647	1.846

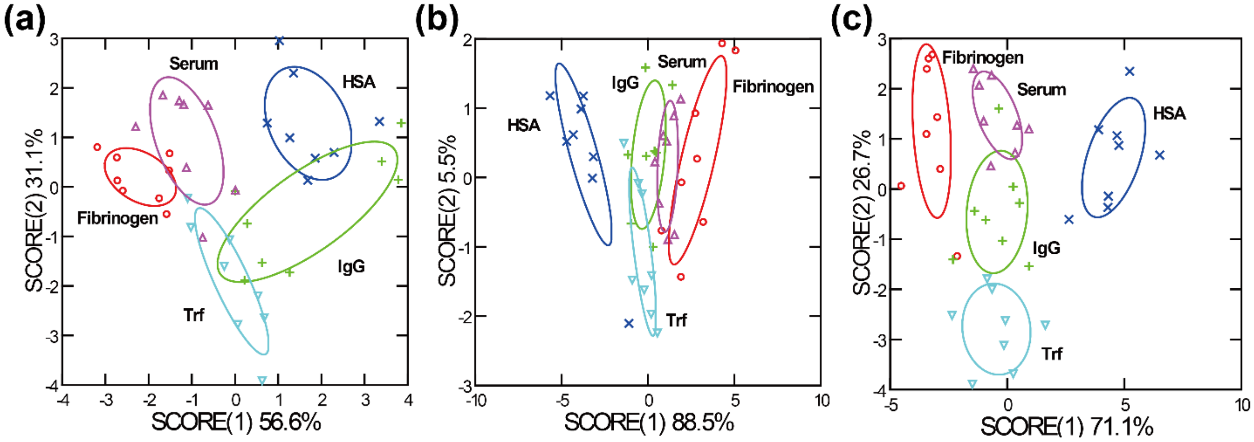

Figure S14. LDA plots generated from fluorescence responses (I/I_0) of PFP (Bz−) and PFP (Bz+) against 10% sera spiked with **a)** 0.4 mg/mL proteins (classification accuracy on the scatterplot: 83%; Jackknife classification: 65%; $n = 8$), **b)** 0.6 mg/mL proteins (classification accuracy on the scatterplot: 80%; Jackknife classification: 63%; $n = 8$), and **c)** 0.8 mg/mL proteins (classification accuracy on the scatterplot: 90%; Jackknife classification: 73%; $n = 8$).

Table S12. Normalized fluorescence responses (I/I_0) and LDA outputs from the sensor array constructed with PFP (Bz−) and PFP (Bz+). Score (1) and (2) correspond to **Figure S13a**.

Sample Name	PFP (Bz−)			PFP (Bz+)			LDA output	
	I/I_0			I/I_0			Score (1)	Score (2)
	Py 1	Py 2	Py Ex	Py 1	Py 2	Py Ex		
Serum	3.392	2.591	1.123	2.558	1.971	1.077	-2.298	1.216
Serum	3.443	2.606	1.067	2.680	2.110	1.136	-1.185	1.666
Serum	3.430	2.590	1.081	2.697	2.098	1.120	-1.289	1.740
Serum	3.447	2.618	1.086	2.639	2.110	1.154	-1.669	1.851
Serum	3.361	2.554	1.045	2.387	1.806	0.955	-0.748	-1.022
Serum	3.363	2.556	1.075	2.635	2.041	1.071	-1.123	0.392
Serum	3.470	2.580	1.029	2.606	1.958	1.041	-0.621	1.650
Serum	3.412	2.583	1.053	2.596	1.937	1.003	0.010	-0.068
Fibrinogen	3.333	2.604	1.128	2.625	2.020	1.081	-1.743	-0.239
Fibrinogen	3.277	2.513	1.063	2.620	2.101	1.146	-3.175	0.794
Fibrinogen	3.292	2.551	1.104	2.557	1.979	1.071	-2.584	-0.087
Fibrinogen	3.331	2.459	0.988	2.242	1.710	0.912	-1.571	-0.561
Fibrinogen	3.342	2.546	1.094	2.613	2.017	1.060	-1.513	0.321
Fibrinogen	3.344	2.486	1.006	2.551	1.906	1.001	-1.500	0.665
Fibrinogen	3.280	2.459	0.964	2.529	1.922	1.043	-2.721	0.584
Fibrinogen	3.232	2.396	1.003	2.475	1.851	0.969	-2.711	0.118
HSA	3.664	2.865	1.183	2.643	1.989	1.071	1.858	0.581

HSA	3.627	2.707	1.112	2.819	2.054	1.070	1.032	2.955
HSA	3.597	2.696	1.101	2.634	1.957	0.990	2.296	0.693
HSA	3.618	2.704	1.076	2.761	2.072	1.082	1.356	2.299
HSA	3.496	2.588	0.988	2.707	2.058	1.048	1.269	0.992
HSA	3.687	2.739	1.084	2.675	2.048	1.034	3.346	1.322
HSA	3.523	2.622	1.036	2.641	1.986	1.033	0.748	1.289
HSA	3.470	2.521	0.924	2.553	1.829	0.908	1.685	0.133
Trf	3.369	2.600	1.127	2.414	1.854	0.963	-0.248	-1.603
Trf	3.319	2.523	1.031	2.609	1.931	0.976	-0.124	-1.068
Trf	3.384	2.669	1.029	2.462	1.922	1.023	0.678	-2.643
Trf	3.355	2.575	1.065	2.460	1.920	1.024	-1.021	-0.818
Trf	3.356	2.510	0.952	2.284	1.651	0.844	0.534	-2.198
Trf	3.287	2.552	1.061	2.463	1.860	0.945	0.066	-2.778
Trf	3.251	2.437	0.946	2.192	1.617	0.794	0.630	-3.916
Trf	3.285	2.389	0.875	2.450	1.859	0.961	-1.095	-0.231
IgG	3.451	2.688	1.157	2.450	1.848	0.965	0.626	-1.533
IgG	3.436	2.588	1.085	2.393	1.746	0.900	0.283	-0.742
IgG	3.432	2.642	1.063	2.500	1.910	0.980	1.265	-1.729
IgG	3.409	2.608	1.102	2.286	1.718	0.896	0.223	-1.888
IgG	3.456	2.615	1.095	2.461	1.894	0.995	0.004	-0.085
IgG	3.764	2.850	1.215	2.406	1.822	0.933	3.778	0.146
IgG	3.779	2.794	1.146	2.222	1.639	0.854	3.400	0.512
IgG	3.714	2.848	1.153	2.923	2.084	1.048	3.852	1.288

Table S13. Normalized fluorescence responses (I/I_0) and LDA outputs from the sensor array constructed with PFP (Bz−) and PFP (Bz+). Score (1) and (2) correspond to **Figure S13b**.

Sample Name	PFP (Bz-)			PFP (Bz+)			LDA output	
	I/I_0			I/I_0			Score (1)	Score (2)
	Py 1	Py 2	Py Ex	Py 1	Py 2	Py Ex		
Serum	3.120	2.448	1.093	2.600	2.058	1.169	0.884	0.602
Serum	3.256	2.575	1.118	2.287	1.833	1.034	1.531	-0.825
Serum	3.134	2.540	1.117	2.470	1.994	1.211	1.497	0.889
Serum	3.238	2.535	1.105	2.507	1.981	1.114	0.398	0.225
Serum	3.015	2.419	1.109	2.591	2.035	1.167	1.085	0.525
Serum	3.106	2.440	1.047	2.532	1.972	1.105	0.646	-0.374
Serum	3.264	2.535	1.002	2.448	1.993	1.141	1.149	-0.895
Serum	3.274	2.436	0.943	2.340	1.791	1.100	1.895	1.133

Fibrinogen	3.086	2.474	1.139	2.200	1.833	1.206	5.091	1.825
Fibrinogen	2.928	2.387	1.305	2.418	1.975	1.164	4.319	1.926
Fibrinogen	3.148	2.541	1.175	2.221	1.785	1.070	2.870	0.263
Fibrinogen	3.000	2.363	1.073	2.462	1.943	1.162	2.768	0.919
Fibrinogen	3.213	2.485	1.051	2.434	1.973	1.138	1.966	-0.077
Fibrinogen	3.194	2.452	0.994	2.287	1.791	1.004	1.933	-1.441
Fibrinogen	3.038	2.306	0.995	2.367	1.855	1.051	3.223	-0.649
Fibrinogen	3.217	2.521	1.043	2.334	1.803	1.025	0.813	-0.772
HSA	3.448	2.666	1.132	2.822	2.165	1.142	-3.867	0.988
HSA	3.336	2.621	1.112	2.820	2.192	1.165	-3.159	0.298
HSA	3.543	2.785	1.150	2.655	2.111	1.127	-3.215	-0.009
HSA	3.663	2.807	1.102	2.947	2.307	1.220	-5.666	1.181
HSA	3.660	2.783	1.073	2.701	2.085	1.114	-4.322	0.618
HSA	3.548	2.634	1.033	2.794	2.121	1.127	-3.736	1.174
HSA	3.524	2.654	1.034	2.789	2.073	1.077	-4.708	0.521
HSA	3.210	2.471	0.951	2.479	1.850	0.969	-1.109	-2.105
Trf	3.279	2.593	1.090	2.438	1.935	1.033	-0.222	-1.624
Trf	3.240	2.529	1.096	2.591	2.023	1.101	-0.560	-0.088
Trf	3.281	2.669	1.104	2.446	1.945	1.076	-0.913	-1.479
Trf	3.305	2.568	1.059	2.656	2.066	1.153	-1.399	0.491
Trf	3.201	2.493	0.984	2.376	1.821	0.989	0.185	-1.971
Trf	3.217	2.410	0.916	2.538	1.965	1.061	0.208	-1.415
Trf	3.531	2.610	1.001	2.365	1.831	1.005	-0.342	-0.237
Trf	3.215	2.402	0.886	2.425	1.858	0.992	0.544	-2.243
IgG	3.333	2.636	1.127	2.507	2.020	1.151	-0.096	0.310
IgG	3.402	2.684	1.181	2.446	1.945	1.142	-0.141	1.585
IgG	3.419	2.715	1.166	2.247	1.830	1.133	1.420	1.333
IgG	3.365	2.581	1.082	2.526	2.051	1.155	0.482	0.357
IgG	3.303	2.592	1.066	2.576	2.113	1.227	0.383	0.381
IgG	3.232	2.467	1.002	2.541	2.002	1.084	0.296	-1.001
IgG	3.437	2.631	1.073	2.502	1.941	1.075	-1.153	0.328
IgG	3.373	2.560	0.997	2.438	1.844	1.006	-0.981	-0.666

Table S14. Normalized fluorescence responses (I/I_0) and LDA outputs from the sensor array constructed with PFP (Bz−) and PFP (Bz+). Score (1) and (2) correspond to **Figure S13c**.

**Sample Name**	**PFP (Bz−)**			**PFP (Bz+)**			**LDA output**	
	**I/I_0**			**I/I_0**			**Score (1)**	**Score (2)**

	Py 1	Py 2	Py Ex	Py 1	Py 2	Py Ex		
Serum	3.431	2.594	1.048	2.679	2.046	1.102	-0.714	0.469
Serum	3.457	2.670	1.098	2.773	2.197	1.178	-1.007	1.344
Serum	3.471	2.749	1.188	2.719	2.092	1.108	0.899	1.214
Serum	3.525	2.745	1.142	2.714	2.049	1.100	0.420	1.268
Serum	3.463	2.668	1.117	2.696	2.067	1.100	0.345	0.702
Serum	3.453	2.733	1.141	2.762	2.149	1.169	-1.215	2.068
Serum	3.768	2.849	1.166	2.633	2.063	1.118	-1.468	2.371
Serum	3.526	2.649	1.103	2.731	2.115	1.148	-0.721	2.269
Fibrinogen	3.199	2.480	1.034	2.430	1.891	1.054	-4.541	0.027
Fibrinogen	3.395	2.679	1.136	2.602	2.036	1.135	-3.449	2.378
Fibrinogen	3.409	2.680	1.142	2.481	1.963	1.077	-3.454	1.074
Fibrinogen	3.473	2.747	1.170	2.582	2.029	1.128	-3.376	2.577
Fibrinogen	3.354	2.713	1.179	2.612	2.040	1.138	-3.224	2.652
Fibrinogen	3.246	2.494	1.011	2.567	2.020	1.077	-2.132	-1.364
Fibrinogen	3.192	2.425	0.994	2.611	2.016	1.111	-2.869	0.395
Fibrinogen	3.286	2.538	1.056	2.642	2.063	1.140	-3.014	1.432
HSA	3.754	2.811	1.099	2.994	2.206	1.141	4.800	0.874
HSA	3.791	2.875	1.115	2.901	2.167	1.104	4.321	-0.339
HSA	3.895	2.945	1.156	2.955	2.246	1.156	3.920	1.192
HSA	3.865	2.937	1.175	3.079	2.335	1.171	6.512	0.739
HSA	3.909	2.913	1.157	3.105	2.360	1.215	5.247	2.343
HSA	3.851	2.922	1.134	3.075	2.345	1.200	4.679	1.103
HSA	3.796	2.811	1.057	2.990	2.248	1.149	4.351	-0.157
HSA	3.746	2.804	1.073	2.804	2.108	1.087	2.660	-0.578
Trf	3.330	2.549	1.020	2.589	2.010	1.052	-0.616	-2.014
Trf	3.340	2.601	1.021	2.641	2.037	1.074	-0.844	-1.798
Trf	0.515	0.886	0.869	2.599	2.000	1.103	-1.451	-3.915
Trf	3.370	2.582	1.042	2.367	1.844	0.973	-2.338	-2.538
Trf	3.234	2.522	1.034	2.551	1.951	1.015	-0.047	-2.657
Trf	3.413	2.567	1.021	2.467	1.896	0.975	-0.118	-3.117
Trf	3.256	2.433	0.953	2.526	1.909	0.984	0.275	-3.681
Trf	3.350	2.493	0.958	2.688	2.002	1.034	1.661	-2.698
IgG	3.323	2.513	1.042	2.759	2.074	1.135	-0.385	1.617
IgG	3.346	2.527	1.034	2.726	2.080	1.108	0.258	0.051
IgG	3.428	2.675	1.085	2.588	1.955	1.035	-0.190	-1.043
IgG	3.300	2.585	1.067	2.677	2.044	1.064	0.949	-1.528

IgG	3.273	2.538	1.040	2.643	2.028	1.085	-0.945	-0.597
IgG	3.471	2.660	1.065	2.391	1.815	0.976	-2.300	-1.425
IgG	3.570	2.716	1.084	2.684	2.051	1.079	0.520	-0.265
IgG	3.481	2.604	1.022	2.630	2.052	1.094	-1.395	-0.444

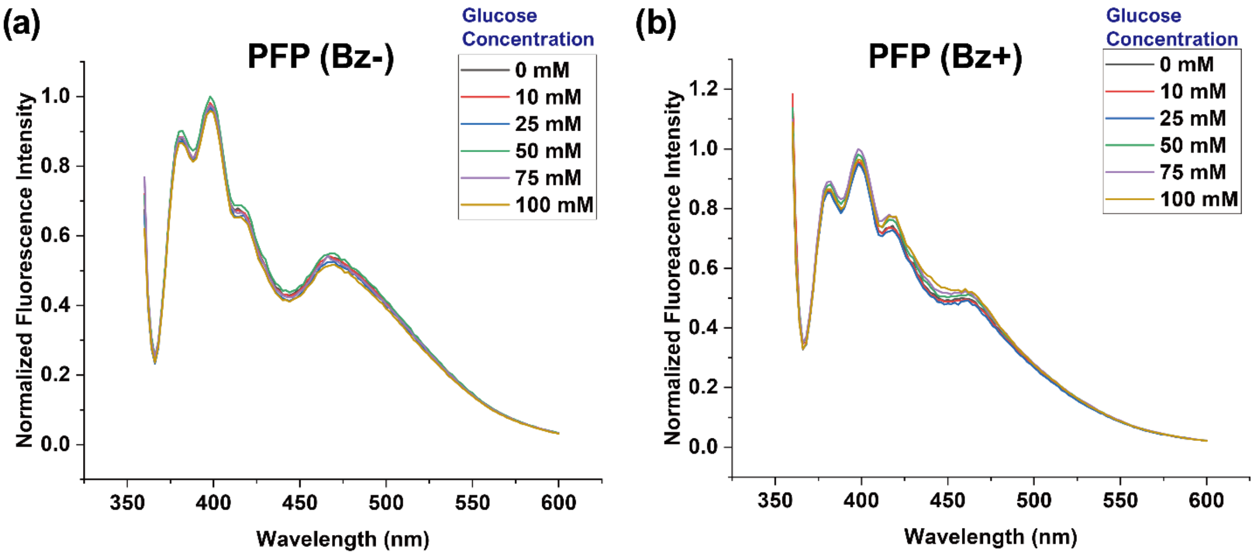

Figure S15. Fluorescence responses of **a)** PFP (Bz−) and **b)** PFP (Bz+) under varying concentrations (n = 3)
